# Environmental Regulation of *Yersinia* Pathophysiology

**DOI:** 10.3389/fcimb.2016.00025

**Published:** 2016-03-02

**Authors:** Shiyun Chen, Karl M. Thompson, Matthew S. Francis

**Affiliations:** ^1^Key Laboratory of Special Pathogens and Biosafety, Wuhan Institute of Virology, Chinese Academy of SciencesWuhan, China; ^2^Department of Microbiology, College of Medicine, Howard UniversityWashington, DC, USA; ^3^Umeå Centre for Microbial Research, Umeå UniversityUmeå, Sweden; ^4^Department of Molecular Biology, Umeå UniversityUmeå, Sweden

**Keywords:** acidity, temperature, metabolism, RovA, c-di-GMP, cAMP, extracytoplasmic stress, transition metals

## Abstract

Hallmarks of *Yersinia* pathogenesis include the ability to form biofilms on surfaces, the ability to establish close contact with eukaryotic target cells and the ability to hijack eukaryotic cell signaling and take over control of strategic cellular processes. Many of these virulence traits are already well-described. However, of equal importance is knowledge of both confined and global regulatory networks that collaborate together to dictate spatial and temporal control of virulence gene expression. This review has the purpose to incorporate historical observations with new discoveries to provide molecular insight into how some of these regulatory mechanisms respond rapidly to environmental flux to govern tight control of virulence gene expression by pathogenic *Yersinia*.

## *Yersinia* biology and classical virulence traits

Pathogenic *Yersinia* have been a long-standing model bacteria for furthering understanding of bacteria-host cell interplay. At center stage for well-over 100 years has been the highly virulent and obligate plague-causing pathogen *Y. pestis*. Having only recently evolved from ancestral *Y. pseudotuberculosis*, a mildly virulent enteric pathogen, has meant that the *Yersiniae* are a model genus to study active pathogen evolution (Wren, 2003; Drancourt, 2012; Rasmussen et al., 2015). Significant evolutionary events in the formation of *Y. pestis* as an obligate pathogen appear to be its genome reduction and the corresponding loss of functional coding potential (Sun et al., 2014; Bolotin and Hershberg, 2015), its re-wiring of regulatory circuitry that permits elevated virulence gene expression during host infections beyond the levels achieved by its close relative *Y. pseudotuberculosis* (Chauvaux et al., 2011; Ansong et al., 2013), as well as the gain of genetic information such as in the form of two additional plasmids pMT1 and pPCP1 encoding the murine toxin and the plasminogen activator, respectively (Chain et al., 2004).

**Figure 1:**
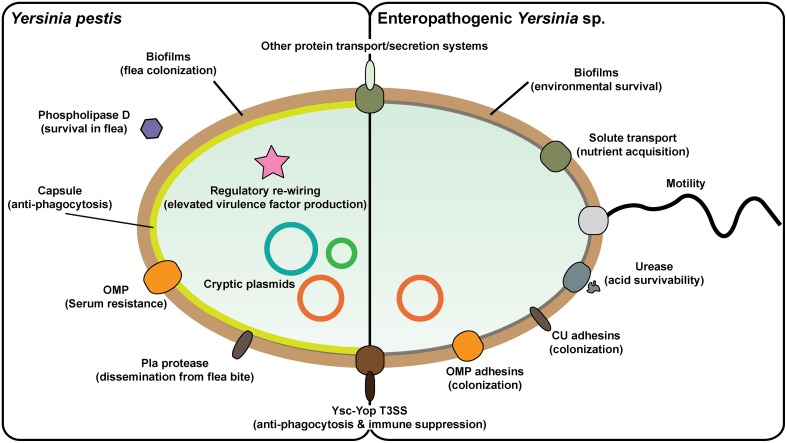
**Prominent *Yersinia* virulence factors**. *Yersinia pestis* and enteropathogenic *Y. pseudotuberculosis* and *Y. enterocolitica* vary greatly in their pathogenicity and in aspects of their pathogenesis. This is reflected by the different repertoire of proven and potential virulence factors in their respective armories. In particular, *Y. pestis* has acquired additional plasmid DNA that encodes for factors that enable colonization and transmission via the flea vector and survival in blood. It is also apparent that the regulatory circuitry of *Y. pestis* has been rewired in ways that drive elevated *in vivo* expression of critical virulence associated factors. On the flip side, *Y. pestis* has lost flagella-mediated motility and cell-adhesive capacities that are otherwise critical for survival of the enterics both in the environment and in the GI tract, respectively. Yet commonalities between all three pathogens exist, such as the prominent virulence plasmid-encoded Ysc-Yop type III secretion system responsible for promoting an extracellular infection niche, along with other systems responsible for distributing *de novo* synthesized proteins into other extracytoplasmic compartments or even realized free from the bacteria.

In many cases, *Y. pseudotuberculosis* and *Y. enterocolitica* can serve as a convenient substitute for the studies of *Y. pestis* pathogenicity and this has meant that much has been learned about the *Yersinia* infectious cycle and how they react to contact with both non-immune and immune cells. Pathogenic *Yersinia* produce numerous surface located proteins that could possess auto-agglutinating properties, engage with host cell surface receptors or act as serum resistance factors that limit the action of complement-mediated opsonization and killing (Figure 1). The most prominent *Yersinia* adhesins studied to date are invasin, YadA, Ail, and pH 6 antigen (Kolodziejek et al., 2012; Zav'yalov, 2012; Mikula et al., 2013; Muhlenkamp et al., 2015). However, their relative importance to the biology of infection is pathogen-dependent, and in certain cases may not be required at all.

*Yersinia* capitalizes on close contact with the host cell to employ a Ysc-Yop type III secretion system (T3SS) for the injection of anti-host effectors into the target cell (Keller et al., 2015; Figure 1). This system is encoded on a virulence plasmid common to all three human *Yersinia* pathogens, and contributes two major virulence traits to *Yersinia*—anti-phagocytic and immunosuppression activities (Plano and Schesser, 2013). Several additional protein secretion systems, especially including a chromosomal T3SS, a T2SS, multiple T5SSs, and T6SSs as well as chaperone-usher systems, are predicted in the genomes of pathogenic *Yersinia* (Yen et al., 2008), and the functionality of some of these have been verified experimentally (Haller et al., 2000; Venecia and Young, 2005; Yen et al., 2007; Felek et al., 2008, 2011; Lawrenz et al., 2009; Robinson et al., 2009; Hatkoff et al., 2012; Lenz et al., 2012; Pisano et al., 2012; Seo et al., 2012; Von Tils et al., 2012; Lane et al., 2013; Walker et al., 2013; Nair et al., 2015; Wang et al., 2015; Figure 1).

Biofilm formation by pathogenic *Yersinia* is another significant virulence trait (Figure 1). The ability of *Y. pestis* to form biofilms in fleas is considered a major evolutionary catalyst by providing a means of bacterial transmission from the flea to mammalian host (Darby, 2008; Hinnebusch and Erickson, 2008; Sun et al., 2014). The *hms* locus is encoded on the chromosome in a high pathogenicity island (HPI) and contributes to virulence of *Y. pestis* and environmental survival of the enteric *Yersinia*. The *hms* locus is responsible for the biosynthesis and secretion of an exopolysaccharide polymer (EPS) matrix material that helps to form highly aggregative biofilm (Darby et al., 2002; Jarrett et al., 2004; Kirillina et al., 2004).

With this knowledge of determinants contributing to bacterial survival in diverse environmental niches, the human pathogenic *Yersinia* represent ideal model systems for studying the environmental regulation of gene expression. *Y. pestis* has a strict lifecycle but still alternates between flea vector and mammalian host, while the food-borne enteropathogens establish environmental niches in soil and water along with intermittent mammalian host infections. As such, this bacterial family encounters many unique environments that all undergo continuous physical and chemical flux. With the capacity to sense this physicochemical flux, pathogenic *Yersinia* respond by utilizing impressive regulatory networks to coordinate the temporal and spatial control of collections of often unlinked genetic loci. This review aims to highlight some of these important sensory and regulatory networks that have capacity to facilitate rapid reprogramming of global gene expression profiles to enable *Yersinia* to adapt, survive, and prosper in selected environmental niches.

## The mainstays of environmental sensing by *Yersinia*

### Responsiveness to acidity

With pH values as low as 1.5–2.5, the acidic environment of the mammalian stomach is a natural barrier against infections of food-borne pathogens. Gastrointestinal bacterial pathogens have thus evolved elaborate mechanisms to cope with excursions into acidic environments. Acid survival mechanisms are remarkably different among different pathogens. To cope with different degrees of acidic environmental stress, several acid survival systems have evolved in enteric bacteria e.g., acid resistance (AR), acid tolerance response (ATR), and acid habituation (AH), and examples of these have been well-documented (Foster, 2004). In particular, at least four acid resistance (AR) systems have been documented. The glucose-repressed AR1 system is controlled by the regulators cAMP receptor protein (CRP) and RpoS (Foster, 2004). The other three AR systems (AR2, AR3, and AR4) are decarboxylase/antiporter-dependent systems that function in pH homeostasis by coupling extracellular glutamate (AR2), arginine (AR3), or lysine (AR4) and their corresponding amino acid decarboxylases GadA/B, AdiA, and CadA, with the cognate antiporters GadC, AdiC, and CadB, respectively (Foster, 2004; Song et al., 2015). The two gastrointestinal pathogens, *Y. pseudotuberculosis* and *Y. enterocolitica* transmit to humans after the ingestion of contaminated water or food. Like many food-borne pathogens, they have developed different survival systems that protect against acidic conditions for successful colonization and infection.

#### Carbohydrate metabolism and acid survival

The role of carbohydrate metabolism in acid survival of enteric bacteria remains largely unknown. Cyclic AMP receptor protein (CRP), which is a hallmark of glucose metabolism regulation, is a regulator of acid survival in *Escherichia coli* (Castanie-Cornet et al., 1999). Significantly, the global transcriptional regulator, Cra (cAMP-independent catabolite repressor/activator), also negatively regulates acid tolerance in *Y. pseudotuberculosis* (Hu et al., 2011). The Cra targets for acid survival regulation remain unknown as does its mechanism of action, but presumably Cra mediates this regulatory role via transcriptional regulation. Further experiments are needed to identify specific regulators to obtain more detailed information about carbohydrate metabolism and acid survival in *Y. pseudotuberculosis*.

#### Amino acid metabolism and acid survival

More is known about the important connections between amino acid metabolism and acid survival in bacteria. Several acid resistance systems, e.g., glutamate-, arginine-, or lysine-dependent, have been described in *E. coli* (Foster, 2004). Notably, several key genes in amino decarboxylase or antiporter-dependent acid resistance systems are absent in the genome of *Y. pseudotuberculosis*, which raises the question of whether other amino acids are involved in acid survival in this bacterium. Indeed, the enzyme aspartate ammonia lyase or aspartase (AspA), which is involved in aspartate metabolism by catalyzing the deamination of L-aspartate to form fumarate and ammonia, plays a role in acid survival in *Y. pseudotuberculosis* (Hu et al., 2010). AspA increases acid survivability of bacteria by producing ammonia from aspartate as demonstrated by mutational and *in vitro* enzyme activity studies. Interestingly, this aspartate-dependent acid survival pathway appears to exist in *Y. enterocolitica* as well as other food-borne pathogenic bacteria including *E. coli* O157:H7 and *Salmonella enterica*, given that the addition of aspartate into culture media also increases their survivability at low pH (Hu et al., 2010). This observation suggests that this enzyme could be a universal mechanism for acid survival of gastric bacteria and might therefore represent a notable target to develop new drugs for the control of bacterial infections. The reasons for why bacteria choose to couple different amino acid utilization pathways with acid responses is unknown, but is certainly worth further investigation.

#### Stress-related proteins and acid survival

The enzyme urease is a major player in the resistance to acidity and plays a central role in colonization and persistence in the host. Consistent with this, urease is constitutively active and comprises between 5 and 10% of the total cellular protein (Stingl and De Reuse, 2005). Urease catalyzes the hydrolysis of urea to yield ammonia, which neutralizes the presence of protons to mitigate acidity (Miller and Maier, 2014). Earlier studies have demonstrated that urease is responsible for an ATR in *Y. enterocolitica* (De Koning-Ward and Robins-Browne, 1995), and a urease mutant of *Y*. *pseudotuberculosis* has lost its ability to survive at pH 3.0 in the presence of urea (Riot et al., 1997; Figure 1). Using comparative proteomic analyses to identify global protein synthesis changes in *Y. pseudotuberculosis* that were induced by growth at pH 4.5 (a sub-lethal pH to this bacterium) compared to neutral pH, further highlighted the importance of urease in acid survival (Hu et al., 2009). Moreover, the OmpR response regulator of the EnvZ/OmpR two-component regulatory system (TCRS), was found to activate urease synthesis to enhance acid survival (Hu et al., 2009). This regulatory control appears to be direct, for the regulatory regions of the multiple urease polycistronic transcriptional units are all individually recognized by specific OmpR binding (Hu et al., 2009).

#### Other features of acid survival in *Yersinia*

Studies on acid survival in *Yersinia* mainly focus on mild acid conditions. Whether and how *Yersinia* survives within an extremely acidic environment (pH < 2.0) remains unclear. As a transcriptional regulator, OmpR may also regulate other acid survival pathways. In a recent study in *Y. pseudotuberculosis*, the production of a thermo-regulated type VI secretion system (T6SS4) was OmpR-regulated, and a direct relationship between this secretion system and an ATR was observed (Zhang et al., 2013a). The involvement of OmpR-regulated T6SS in pH homeostasis and acid tolerance is through proton efflux, and is dependent upon the ATPase activity of ClpV4—a core component of all T6SSs—that participates in proton extrusion (Zhang et al., 2013a). However, whether the T6SS4 is directly acting as a proton transporter remains obscure. Nevertheless, this is a novel acid survival strategy in which a protein secretion system associated with virulence has also an unexpected role in proton extrusion under acid conditions.

In *Y. pseudotuberculosis*, Song et al. recently demonstrated that RovM, a central regulator of the CsrABC-RovM-RovA cascade, inversely regulates two established acid survival systems (Song et al., 2015). In particular, RovM bound the promoters of T6SS4 genes to activate T6SS4 synthesis, but also bound to the −35 element in the arginine-dependent acid resistance system (AR3) promoter to repress AR3 synthesis. The authors proposed that RovM coordinately regulates the production of AR3 and T6SS4 in response to the availability of nutrients in the environment (Song et al., 2015).

In addition to OmpR, other TCRSs have also been reported to play a role in acid stress responsiveness by *Yersinia*. The response regulator PhoP of the PhoP-PhoQ TCRS is necessary for survival of *Y*. *pseudotuberculosis* in macrophages (Grabenstein et al., 2004; Bozue et al., 2011). These data were corroborated by a systematic analyses of two-component regulons in *Y. pseudotuberculosis* in which acid responsiveness depended upon intact *phoP-* and *ompR*-dependent regulatory pathways, as well as noting an involvement of the *pmrA-*dependent regulatory pathway (Flamez et al., 2008). The *phoP* gene of *Y. pestis* is also required for intracellular survival in macrophages and depending on the transmission route, also for virulence (Oyston et al., 2000; O'loughlin et al., 2010; Bozue et al., 2011). However, rather than being involved in acid responsiveness *per se*, it seems most likely that *Y. pestis* PhoP actually regulates essential survival genes (Grabenstein et al., 2006; O'loughlin et al., 2010) perhaps through its well-known ability to sense magnesium ions (Zhou et al., 2005; Li et al., 2008). Finally, the twin arginine translocation (Tat) pathway, which is essential for bacterial virulence, has also been demonstrated to contribute to acid survival in *Y. pseudotuberculosis* (Lavander et al., 2006). Though different acid survival systems have been documented in *Yersinia*, the molecular mechanisms integrating the coordination of these are not fully understood. No doubt an increase knowledge of acid survival mechanisms could benefit possibilities to develop broad-spectrum novel strategies for the prevention and treatment of infections by *Yersinia* and other food-borne pathogens.

#### Acid response systems in *Yersinia pestis*

*Y. pestis* does not utilize the intestinal route of infection, but is still likely to encounter acidic environments during the infectious process, especially upon internalization by immune cells (Lukaszewski et al., 2005; Spinner et al., 2014). Hence, it is anticipated that *Y. pestis* would require acid responsive systems for survival during infections. Indeed, *in silico* analyses suggests all known acid survival systems are complete and intact in *Y. pestis*, except for the established loss of urease activity (Table 1). Actually, all *Y. pestis* strains contain a mutated *ureD* gene that abolishes urease activity, and this is considered to be a key evolutionary step that facilitated the adaptation of *Y. pestis* to the flea-borne transmission route (Chouikha and Hinnebusch, 2014). Interestingly, the AR3 system is widespread in all three human pathogenic *Yersinia*, and these represent general acid survival/tolerance systems. However, no obvious acid survival related metabolic protein or AR system seems to be solely *Y. pestis* specific, which is consistent with the idea that these bacteria promote intracellular survival by dedicated mechanisms that avoid acidification of the *Y. pestis-*containing vacuoles (Pujol et al., 2009). By extension, the AR2 system is present only in *Y. enterocolitica*, and is most probably important for safe passage of these bacteria through the acidified environment of the mammalian stomach, thus offering an explanation as to why this bacterium seems to be more resistant to acid environments than is *Y. pseudotuberculosis* (Hu et al., 2010).

**Table 1:** **Comparison of acid survival and tolerance systems in all three human pathogenic *Yersinia***.

**Acid survival systems**	***Y. enterocolitica***	***Y. pseudotuberculosis***	***Y. pestis***
**METABOLIC FACTORS**			
Cra	✓	✓	✓
OmpR	✓	✓	✓
PhoP	✓	✓	✓
RovM	✓	✓	✓
Urease	✓	✓	Non-functional due to *ureD* mutation
**AR2 SYSTEM**			
GadA	✓	✘	✘
GadB	✓	✘	✘
GadC	✓	✘	✘
**AR3 system**			
AdiA	✓	✓	✓
AdiC	✓	✓	✓
**AR4 SYSTEM**			
CadA	✓	✘	✘
CadB	✘	✘	✘
ClpV4	✘	✓	✓

*✘: not detected (absent). ✓: present and genetically intact*.

#### Alkalinity and Na^+^/H^+^ antiport

Precious little information describes the adaptation of *Yersinia* to alkaline environments. However, *in silico* evidence indicates that *Y. pestis* encodes the capacity to couple sodium ion cycling to energy metabolism, and this would constitute a bacterial adaptation strategy to maintain pH homeostasis particularly when exposed to alkaline environments (Hase et al., 2001; Mulkidjanian et al., 2008; Ganoth et al., 2011). To begin to understand the role of sodium ion cycling in *Y. pestis* physiology, Minato and colleagues established knockouts of the loci encoding the primary Na^+^ ion pump, NQR, and the secondary Na^+^ ion pumps known as the NhaA and NhaB Na^+^/H^+^ antiporters (Minato et al., 2013). They found that *Y. pestis* lacking both antiport systems were attenuated demonstrating clearly a role for Na^+^/H^+^ antiport in virulence. Interestingly, the NhaA Na^+^/H^+^ antiporter activity is pH dependent with maximal activity exhibited in alkaline conditions (Ganoth et al., 2011). Taken together, this suggests that the obligate lifecycle of *Y. pestis* demands that it has the potential to adapt to alkaline environments, and at least one of these adaptation mechanisms is via the NhaA antiport system.

### Global effects of temperature

Temperature varies widely as microbes alternate between environmental, invertebrate, and/or vertebrate reservoirs. It is therefore commonly sensed by pathogens to recognize their environment and control virulence gene expression. Multiple molecular mechanisms of temperature-dependent control are well-described in the literature. In *Yersinia, two* prominent thermally controlled virulence properties are the plasmid encoded Ysc-Yop T3SS and the global regulator RovA.

#### Thermoregulation of the Ysc-Yop T3SS

**Figure 2:**
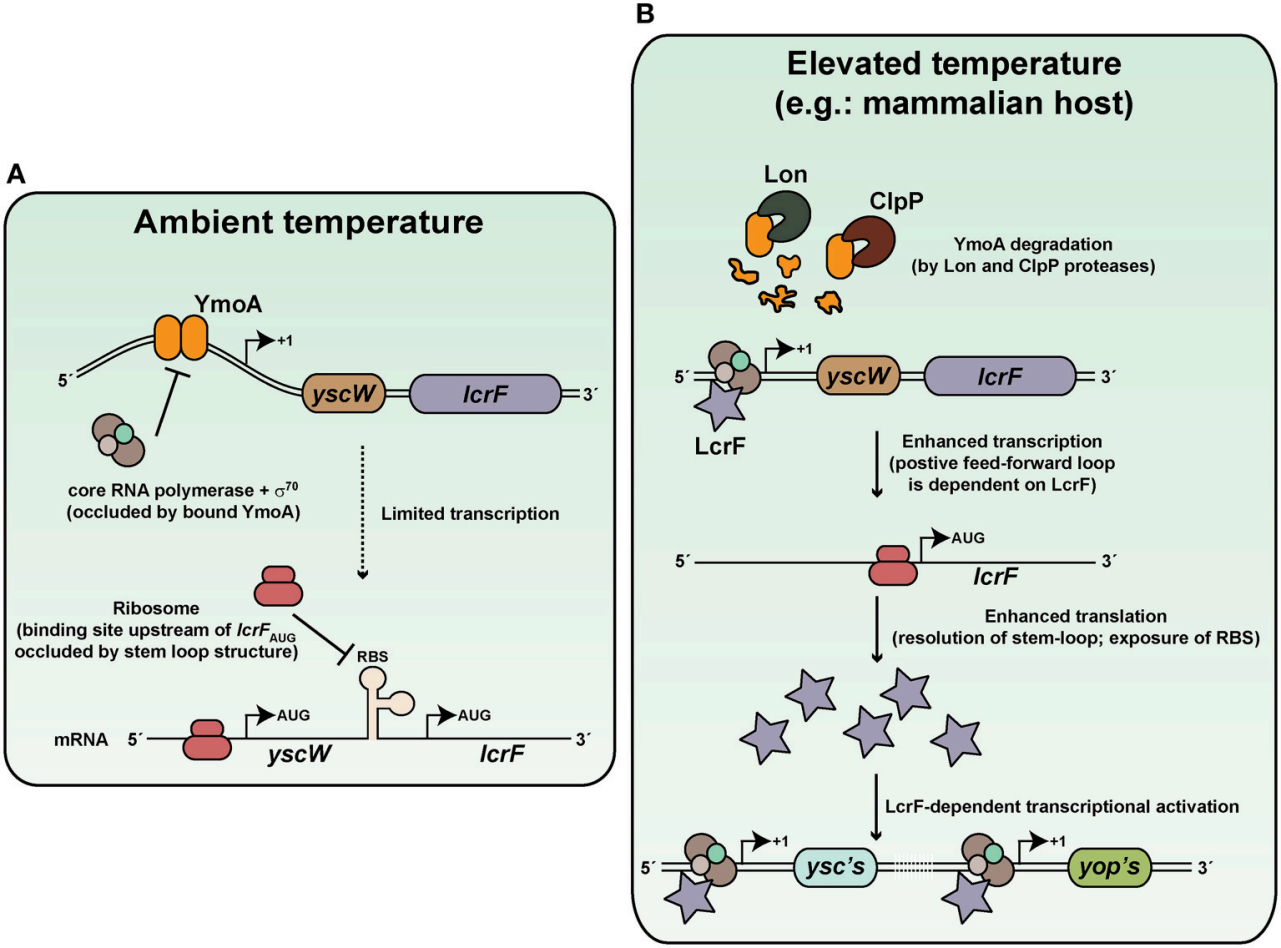
**Thermoregulation of Ysc-Yop type III secretion by *Yersinia***. Thermoregulation of Ysc-Yop type III secretion is mediated through control of the transcriptional activator, LcrF. **(A)** At ambient temperature, transcription of the yscW-lcrF operon is inhibited by the YmoA DNA binding protein. Furthermore, post-transcriptional inhibition occurs as a result of stem-loop formation of mRNA within the intergenic region between the two alleles. **(B)** De-regulation occurs at elevated temperature because YmoA affinity for the *yscW-lcrF* operon promoter is dramatically diminished, and this promotes operon transcription. Moreover, elevate temperature resolves the stem-loop structure in mRNA transcripts, so that translation into LcrF can proceed. This results in a positive auto-regulatory cascade that enhances lcrF transcription. Accumulated LcrF can then transcriptionally activate responsive ysc and yop promoters. This illustration is inspired in part from Bohme et al. (2012) and initial artistic work of Tiago Costa.

It has been known for many years that temperature upshift from ambient temperature to 37°C and target cell contact are crucial to triggering Ysc secretion apparatus synthesis and the subsequent translocation of its specific cargo (Rosqvist et al., 1994; Persson et al., 1995; Pettersson et al., 1996). A cornerstone of this temperature response involves the AraC-like transcriptional activator, LcrF (Yother et al., 1986; Cornelis et al., 1989; Hoe et al., 1992; Lambert De Rouvroit et al., 1992; Hoe and Goguen, 1993; Figure 2A). Through a C-terminal helix-turn-helix motif, LcrF binds to DNA sequences overlapping the −35 region of σ^70^-dependent promoters to auto-activate *lcrF* expression as well as activate the expression of other *ysc* and *yop* genes (Wattiau and Cornelis, 1994; King et al., 2013). At ambient temperature, the DNA architecture of the *lcrF* promoter is conducive to binding by the small nucleoid associated protein YmoA and this impedes transcriptional output (Cornelis et al., 1991; Bohme et al., 2012). Furthermore, the presence of a complex stem loop structure in the 5-prime untranslated region of *lcrF* mRNA transcripts conceal the Shine-Dalgarno sequences from the ribosome to prevent its translation (Bohme et al., 2012). However, an elevation in the surrounding temperature sees a conformational change in the DNA curvature encompassing the *lcrF* promoter (Rohde et al., 1994, 1999) and also in the product of its transcription (Hoe and Goguen, 1993; Bohme et al., 2012; Figure 2B). Consequently, YmoA binding affinity is diminished and this free protein is degraded by the ClpP and Lon proteases (Jackson et al., 2004; Bohme et al., 2012). This allows RNA polymerase holoenzyme access to the *lcrF* promoter, and in collaboration with LcrF, will dramatically enhance transcription. In parallel, stem-loop structures in the *lcrF* mRNA denature, and this establishes ribosomal recognition and subsequent LcrF translation (Hoe and Goguen, 1993; Bohme et al., 2012). In this way, at least two levels of thermo-control regulate *lcrF* expression, which in turn impacts on the ability to synthesize Ysc proteins for assembly into a T3SS.

However, recent research has demonstrated that LcrF levels are directly controlled by additional regulatory factors that include the IscR iron-sulfur cluster regulator (Miller et al., 2014), the LysR-like transcriptional regulator YtxR (Axler-Diperte et al., 2009), CpxR of the CpxA-CpxR two-component system (Carlsson et al., 2007a; Liu et al., 2012) and RscB of the Rsc phosphorelay system (Li et al., 2015). Thus, despite *Yersinia's* responsiveness to temperature fluctuations having a major impact on Ysc-Yop T3SS control, other distinct regulators are clearly required to enable these bacteria to further fine-tune LcrF production in response to additional environmental cues. The precise interplay between these different regulators has not been investigated. However, it seems that YtxR functions to prevent engagement of a positive feed-forward loop by competing with LcrF for overlapping binding sites within target promoters in *Y. enterocolitica* (Axler-Diperte et al., 2009).

#### Thermoregulation of RovA

**Figure 3:**
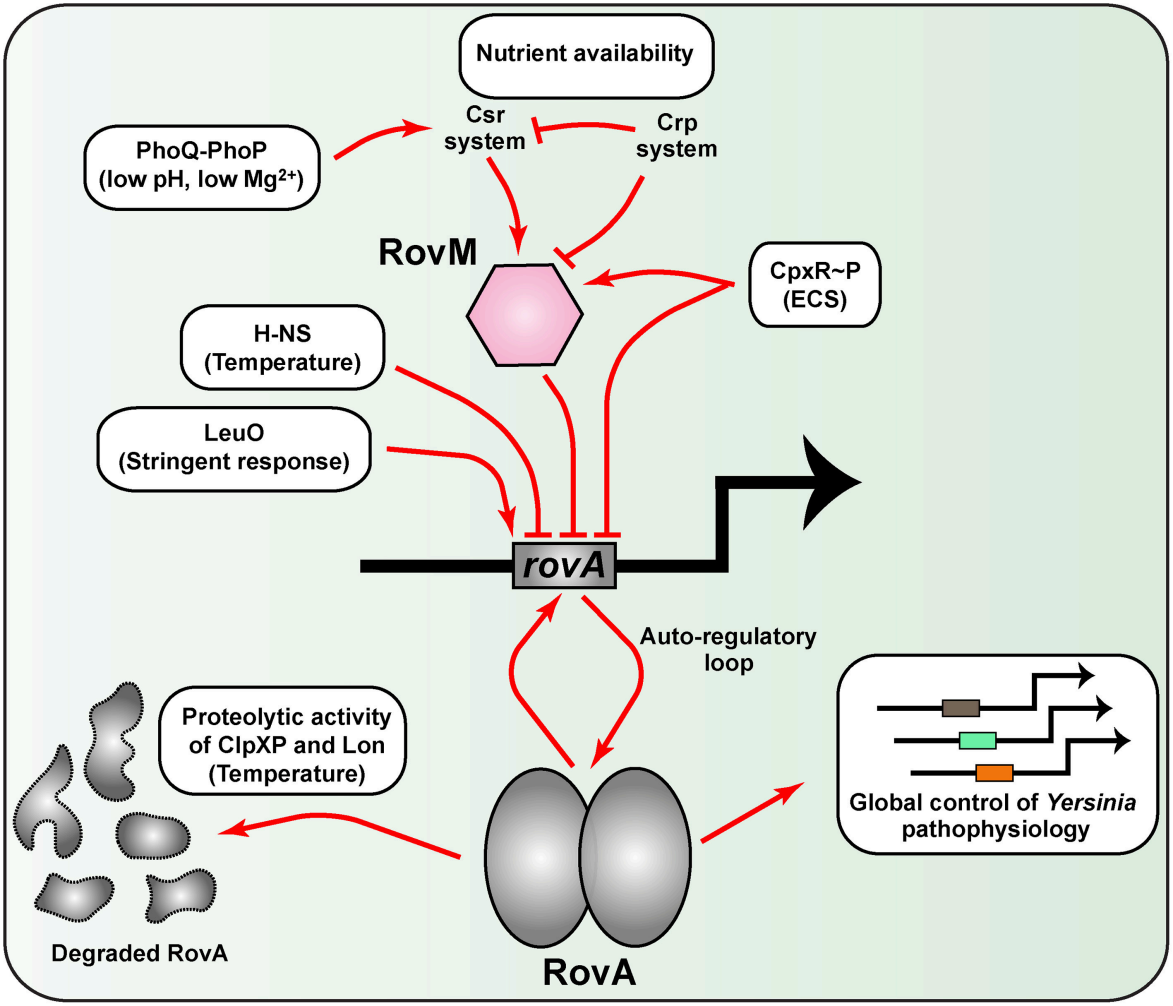
**A network of diverse regulatory inputs controls RovA transcriptional output in *Yersinia***. Available RovA is strictly controlled by cascade regulation at both the transcriptional and post-transcriptional levels in response to multiple environmental cues. The strongest influence on RovA production is through two opposing pathways. The first is an auto amplification loop, which in turn is responsive to thermo-regulated proteolysis of RovA by ClpXP and Lon proteases. The second is via RovM that is principally mediated by the prominent Csr and Crp pathways responsive to carbon and glucose availability. Other regulatory pathways are known, but the extent to which they alter RovM or RovA levels is less clear. In the diagram, induction of RovA expression is indicated by an arrow, while repression is indicated by a blunted line. For simplicity, information concerning whether the pathway is direct or indirect has been omitted on the basis that this is not always defined.

A second example of thermoregulation involves RovA, a MarR-type dimeric winged-helix DNA-binding protein (Ellison and Miller, 2006b). RovA is a master regulator of several physiological properties of pathogenic *Yersinia*, including metabolic and stress adaptation as well as virulence (Cathelyn et al., 2007; Yang et al., 2010). Transcription of *rovA* is positively auto-regulated in a temperature-dependent manner (Heroven et al., 2004; Zhang et al., 2011), while repression occurs largely through the DNA binding elements H-NS and/or YmoA in complex with RovM out-competing RovA for binding within the extended regulatory region upstream of *rovA* (Heroven et al., 2004; Tran et al., 2005; Ellison and Miller, 2006a; Figure 3). Intriguingly, thermoregulation of RovA occurs post-translationally, and is mechanistically defined by elevated temperature imparting intrinsic structural changes in the RovA homodimer that then specifically limits target DNA binding (Herbst et al., 2009; Quade et al., 2012). In turn, these temperature-induced conformational changes renders RovA more susceptible to proteolytic processing by the ClpP and Lon proteases (Herbst et al., 2009). Critically, thermosensing is inherent to the structural properties of RovA, for the close relative SlyA is thermotolerant (Quade et al., 2012). Hence, *Yersinia* has adapted a global regulator into a unique protein thermosensor that presumably affords these bacteria the capacity to rapidly adapt both environmental survival and virulence properties to the prevailing temperature conditions. However, consistent with its prominent role in coordinating multiple *Yersinia* physiological functions, the need to keep RovA levels closely checked has resulted in the integration of additional cues such as nutrient availability, bacterial growth phase and extracytoplasmic stress responsiveness (Heroven and Dersch, 2006; Heroven et al., 2008; Liu et al., 2011; Nuss et al., 2014). Some interesting aspects of these regulatory features are discussed later on in this review.

## Survival in noxious environments

**Figure 4:**
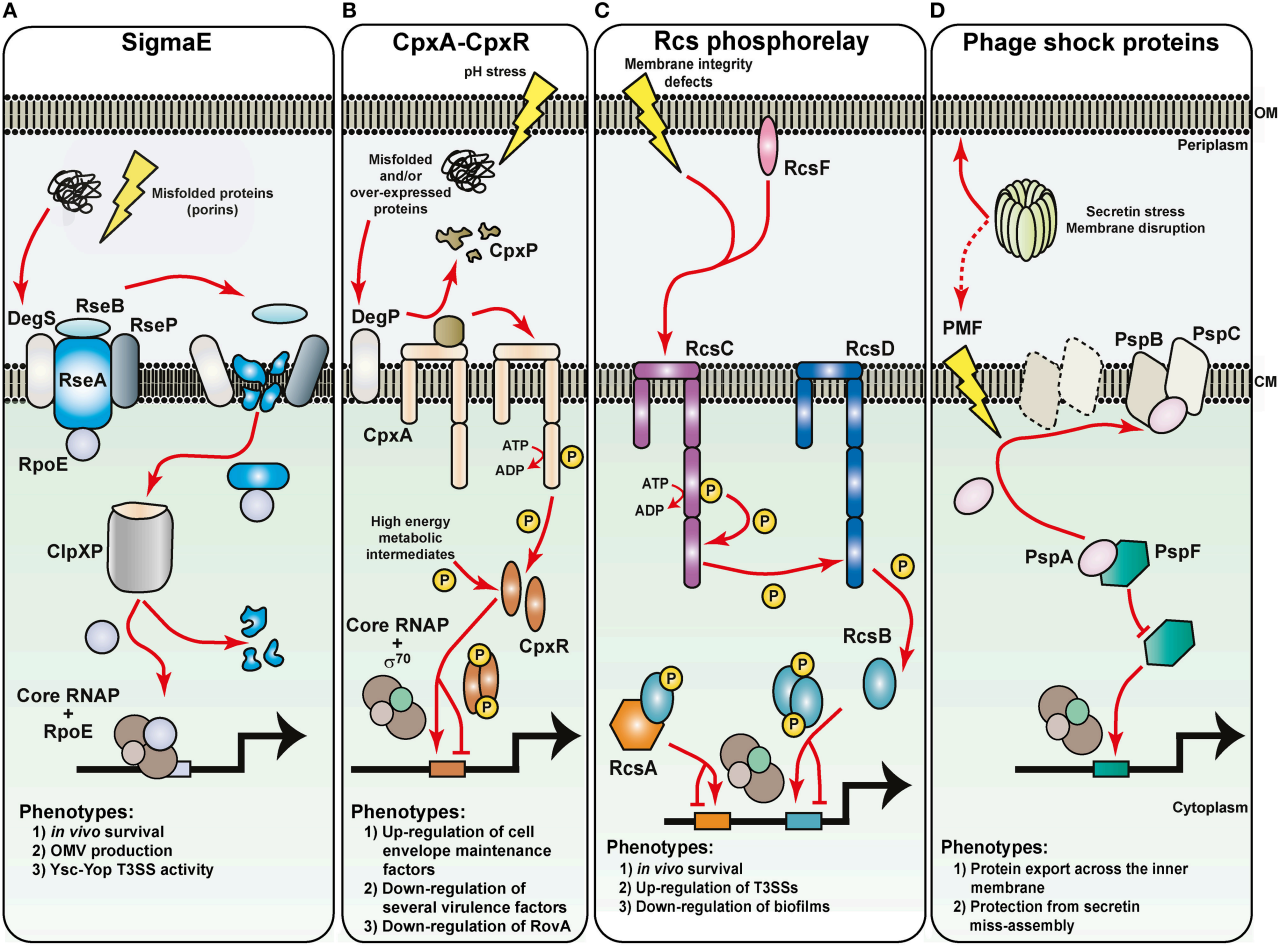
**Sensing of noxious extracytoplasmic stress by the bacteria envelope of *Yersinia***. The molecular basis for the activation of four prominent extracytoplasmic stress sensing sentinels are displayed, along with a summary of their respective phenotypic effects in pathogenic *Yersinia*. Maintaining the outer membrane (OM) are the RopE-, Cpx-, and Rcs-pathways. **(A)** Outer membrane protein misfolding initiates digestion of the anti-RpoE factor, RseA, through successive proteolytic actions of the DegS, RseP, and ClpXP proteases. Free RpoE is released into the cytoplasm, and when engaged with core RNA polymerase (RNAP), can establish controlled transcription of a large RpoE-regulon. The Cpx two-component system **(B)** and the Rcs phosphorelay system **(C)** both rely upon sensor kinase autophosphorylation (CpxA and RscC, respectively) to initiate the transduction of phosphate through to the cytoplasmic cognate response regulator (CpxR and RcsB, respectively). CpxA activation requires the DegP-dependent release of inhibitory CpxP, while RcsC activation might utilize an RcsF-dependent pathway. Inorganic phosphate can also be donated from unstable high-energy metabolic intermediates. Active phosphorylated response regulators dimerize and in concert with the house-keeping RNAP holoenzyme, target specific responsive promoters to influence transcriptional output. RcsB transcriptional control sometimes requires partners with RcsA. **(D)** Controlling the integrity of the cytoplasmic membrane (CM) are the phage shock proteins (psp). Secretin complex mislocalization to the cytoplasmic membrane risks dissipating the proton motive force (PMF). This is prevented by the PspB and PspC proteins actively sequestering the anti-PspF factor, PspA, to free up the PspF transcription factor to initiate promoter targeting and transcriptional output by the house-keeping RNAP holoenzyme.

Protein content in the membrane of Gram negative bacteria contributes important patho-physiological functions necessary for viability. Exposure of membrane to damaging agents therefore poses a significant threat to bacterial survival, for when membrane integrity is compromised, so too is protein transport, and their folding and assembly in these compartments. To circumvent this, bacterial exposure to membrane damaging agents induce physiological responses that are termed extracytoplasmic stress (ECS) responses that ultimately serve to maintain the integrity of the bacterial envelope entity and ensure that the proteins residing in these compartments are functionally able to sustain life. The five known pathways responsive to ECS are the Bae, Cpx, Psp, Rcs, and σ^E^ pathways, most of which are well-characterized especially in *E. coli* (Figure 4). Crucially, accumulating evidence indicates that ECS responsiveness via some of these pathways are also a major regulator of bacterial virulence gene expression, and this is true of pathogenic *Yersinia* (Flores-Kim and Darwin, 2014).

### σ^E^-dependent cell envelope stress response

Arguably the most prominent sentinel of ECS is the σ^E^ (RpoE) pathway, which is also a model for regulated proteolysis (Barchinger and Ades, 2013; Guo and Gross, 2014; Paget, 2015). Reflecting this prominence, the σ^E^-dependent cell envelope stress response in *E. coli* boasts the largest regulon of all the five pathways (Bury-Mone et al., 2009). The key players in this pathway are the transcription factor σ^E^, the inner membrane-located anti-sigma factor RseA and RseB, the inner membrane proteases DegS and RseP and the cytoplasmic protease ClpXP (Figure 4A). In unstressed cells, a complex of RseA-RseB binds σ^E^. Upon exposure to ECS, damaged intermediates that fall off the outer membrane protein assembly pathway accumulate in the periplasm, where they are sensed by, and activate the inner membrane protease DegS. Activated DegS forces RseB to detach from RseA, exposing the periplasmic domain of the latter to DegS-mediated proteolytic digestion. The remaining transmembrane portion of RseA then becomes a target for further digestion by the second inner membrane protease RseP. Now released into the cytoplasm, a soluble complex of σ^E^ bound to the cytoplasmic domain of RseA is specifically targeted by adapter proteins to the molecular chaperone ClpXP. ClpXP-mediated digestion of RseA enables release of σ^E^. Free σ^E^ then competes with other sigma factors for core RNA polymerase (RNAP). The RNAP-σ^E^ holoenzyme then activates expression of the σ^E^ regulon that involves very many factors that contribute to the transport, assembly, and turn-over of outer membrane proteins.

In *Yersinia*, the *rpoE* gene is either essential (Heusipp et al., 2003) or essential for growth upon exposure to stress (Palonen et al., 2013). It follows that *rpoE* expression is inducible in *Y. enterocolitica* during a mouse model of infection (Young and Miller, 1997), as are known σ^E^-regulon members when these bacteria are exposed to intracellular stress following phagocytosis by macrophages (Yamamoto et al., 1997). This indicates that RpoE might be responsible for controlling the synthesis of certain *Yersinia* determinants especially needed for *in vivo* survival (Figure 4A). This is not without precedent for the idea of a virulence specific regulatory role for *rpoE* in bacterial pathogens has been suggested already based upon analyses of *in silico* data (Rhodius et al., 2006). Consistent with this idea, genetically elevating σ^E^ levels via the removal of the anti-σ^E^ regulator, RseA, from *Y. pestis* resulted in the over-production of outer membrane vesicles (Eddy et al., 2014) that in other bacteria are a known contributor to pathogenicity (Ellis and Kuehn, 2010; Avila-Calderon et al., 2015). Using a similar genetic approach in *Y. pseudotuberculosis*, it was demonstrated that σ^E^ accumulation enhanced the synthesis of the virulence plasmid encoded Ysc-Yop T3SS (Carlsson et al., 2007a). Moreover, a gene deletion of *rpoE* resulted in lower production of Ysc-Yop components (Carlsson et al., 2007a; Figure 4A). Since *ysc-yop* gene expression is dependent on the housekeeping RNAP-σ^70^ holoenzyme, the effect of σ^E^ on Ysc-Yop production is probably indirect and points toward the possible involvement of one or more periplasmic protein quality control factors in the assembly of an Ysc-Yop T3SS in the *Yersinia* cell envelope. Thus, together these studies point toward vital regulatory roles of σ^E^ in pathogenic *Yersinia* that are central for ensuring survival under both extracellular environmental stress and intracellular stress.

### Cpx two-component pathway

The CpxA-CpxR system is a classic TCRS, and responds to ECS (Hunke et al., 2012; Raivio, 2014). CpxA is located in the inner membrane and possesses auto-kinase activity. Additionally, it is both a kinase and phosphatase to the cognate CpxR response regulator located in the cytoplasm (Figure 4B). A third component, the periplasmic located CpxP, is responsible for interacting with CpxA to mediate ECS signal recognition (Tschauner et al., 2014). Upon sensing ECS, CpxA is freed from the clutches of CpxP, which is subsequently degraded by the DegP protease. CpxA then auto-phosphorylates and the phosphate group is readily passed on to CpxR. Active CpxR (CpxR~P) then acts as a transcription factor to activate or repress in the vicinity of 100 gene targets in non-pathogenic laboratory *E. coli* (Bury-Mone et al., 2009; Price and Raivio, 2009) or the pathogens *Haemophilus ducreyi* and *Vibrio cholera* (Labandeira-Rey et al., 2010; Gangaiah et al., 2013; Acosta et al., 2015). As ECS causes protein misfolding in the periplasm, this is counteracted by CpxR~P activating the production of protein folding and degradation factors that are destined to exert their function in the periplasm on these misfolded proteins. Additional regulon members also include LPS and phospholipid biosynthesis and transport operons. These are turned on by an active CpxR~P, which serves to maintain and enhance membrane integrity and barrier function. The Cpx pathway therefore functions to optimize bacterial fitness in harsh growth environments, and is a role conserved in many Gram negative bacteria (De Wulf et al., 2000).

Extensive work with this system in *Y. pseudotuberculosis* confirms the sentinel role of CpxR~P in maintaining cell envelope integrity. More significantly however, it was demonstrated that high CpxR~P levels accumulate in mutants devoid of CpxA phosphatase activity—most probably via the indiscriminate action of certain metabolic intermediates that can act as phosphodonors (Liu et al., 2011, 2012)—to repress gene transcription of essential *Yersinia* virulence determinants including the surface adhesin invasin and the plasmid encoded Ysc-Yop T3SS (Carlsson et al., 2007a,b; Liu et al., 2012; Figure 4B). This repression can be direct, for CpxR~P is able to bind to the promoter regions of the *inv* gene (encoding for invasin) and the *rovA* gene (encoding for the positive regulator of *inv* expression; Carlsson et al., 2007a,b; Liu et al., 2012) as well as to the promoter regions of numerous *ysc-yop* encoding operons including *lcrF* (encoding the AraC-like transcriptional activator of *ysc-yop* gene expression; Liu et al., 2012). As a consequence, CpxR~P accumulation leads to an acute reduction in *Y. pseudotuberculosis* toxicity toward infected human tissue culture cell lines (Carlsson et al., 2007b; Liu et al., 2011). Hence, it appears that the CpxA-CpxR signaling pathway functions in sensing noxious ECS to maintain envelope integrity while repressing virulence gene expression; processes crucial to *Yersinia* survivability in extreme environments.

With the ability to repress a large array of virulence determinants in several different bacterial pathogens, it is not surprising that genetically activated Cpx signaling results in virulence attenuation of bacteria in various *in vivo* infection models (Humphreys et al., 2004; Herbert Tran and Goodrich-Blair, 2009; Spinola et al., 2010; Leuko and Raivio, 2012; Debnath et al., 2013; Bontemps-Gallo et al., 2015; Thomassin et al., 2015). Yet, it remains to be seen if a fully intact Cpx system is actually activated in the *in vivo* environment of a eukaryotic host. After all, the primary role of the Cpx signaling pathway might be in free-living bacterial populations exposed to the naturally occurring membrane-damaging elements, and where the production of virulence determinants is seldom required.

Bacteria utilize cascade regulation to establish robust regulatory networks for the tight control of gene expression. Recent work indicates that Cpx signaling affects the levels of other regulatory factors, including non-coding regulatory RNAs, creating integrated regulatory networks (Vogt et al., 2014). In all three human pathogens of *Yersinia*, the RovA molecule is a key global regulator of gene expression (Cathelyn et al., 2006, 2007). The regulation of RovA is complex and involves autoregulation as well as global regulatory factors that sense products of central metabolism including the carbon storage regulatory system (Csr), cAMP receptor protein (Crp), and RovM (Heroven et al., 2008). Moreover, under genetically manipulated conditions where CpxR~P is known to accumulate to high levels, it was shown that CpxR~P also targets the *rovA* promoter and represses transcriptional output (Liu et al., 2011; Figure 4B). This suggests a scenario whereby *Yersinia* has evolved a network of integrated regulatory elements that work together to coordinate gene expression in response to both nutrient availability and ECS.

### Rcs phosphorelay pathway

The complex Rcs signal transduction phosphorelay system is largely exclusive to the Enterobacteriaceae family (Majdalani and Gottesman, 2005; Huang et al., 2006). Membrane bound RcsC is the sensor kinase that upon auto-phosphorylation then transfers the phosphoryl group to the membrane-bound intermediate phosphotransfer protein RcsD, which then transfers it to RcsB, the response regulator. Phosphorylated RcsB monomers dimerize to establish binding to promoter targets, but this may instead require a monomer to first heterodimerize with the auxiliary protein RcsA. An outer membrane lipoprotein-like factor, RcsF, can also stimulate Rcs phosphorelay, but many RcsF-independent inputs also exist (Figure 4C). Most inputs are likely to have resulted from disruptions to cell envelope integrity or alterations in its composition (Majdalani and Gottesman, 2005; Huang et al., 2006). First identified as a regulator of capsule biosynthesis, it is now recognized as an important regulator of diverse processes such as cell division, flagella biosynthesis and motility, small regulatory RNA biosynthesis, biofilm formation, and pathogenicity (Majdalani and Gottesman, 2005; Huang et al., 2006).

The *rcs* loci are present in all *Yersinia* species, but in *Y. pestis* the *rcsA* allele is non-functional and the *rcsD* allele is frameshifted but a functional product is produced (Hinchliffe et al., 2008; Sun et al., 2008). For this reason, initial studies of the Rcs phosphorelay system focused mainly on the enteropathogenic *Yersinia* to reveal an important role in bacterial survival when grown under a variety of environment stresses (Hinchliffe et al., 2008) or during the initial stages of gastrointestinal colonization in a murine model of infection (Venecia and Young, 2005; Figure 4C). Consistent with this, transcriptional profiling indicated an extensive Rcs regulon in *Y. pseudotuberculosis* with many targets functionally linked to the bacterial envelope or in survival within the host or when free-living in the environmental (Hinchliffe et al., 2008). Thus, the finding that RcsB positively regulates the plasmid-encoded *ysc-yop* T3SS genes in *Y. pseudotuberculosis* (Li et al., 2015; Figure 4C) is significant for it establishes a direct involvement of the Rcs system as a player in systemic infections when *Yersinia* is in direct contact with phagocytic immune cells. Interestingly, the Rcs system also positively influences the chromosomal-encoded *ysa-ysp* T3SS genes that play an important role during the early gastrointestinal stage of murine infections by *Y. enterocolitica* (Venecia and Young, 2005). Control of *ysa-ysp* expression is further complicated by the involvement of a second regulatory element, the Ysr phosphorelay system, which ironically might be analogous to the Rcs system (Walker and Miller, 2004, 2009). This suggests that the Rcs and Ysr systems can respond to distinct environmental niches to impart spatial control on virulence gene expression as a strategy to promote both initial colonization and also systemic dissemination into deeper tissue. A deeper understanding of how these pathways coordinate the regulatory events in the different bacteria will therefore provide valuable insight into key aspects of enteric *Yersinia* pathogenicity.

In highly virulent *Y. pestis*, the ability to form biofilms is a key in the initial colonization of the flea foregut and the subsequent flea-borne transmission of this pathogen. Initially, it had been observed that the Rcs pathway is a inhibitor of biofilm in both *Y. pestis* and *Y. pseudotuberculosis* (Sun et al., 2008). Subsequent studies have revealed that a complex of RcsAB is a direct repressor of *Yersinia* biofilm by targeting the *hmsCDE, hmsT*, and *hmsHFRS* loci that encode for enhancers of biofilm development (Sun et al., 2012; Fang et al., 2015; Guo et al., 2015; Figure 4C). Both HmsD and HmsT are diguanylate cyclases required for c-di-GMP production (Bobrov et al., 2011; Sun et al., 2011), an activity enhanced by the regulatory function of HmsC and HmsE (Ren et al., 2014; Bobrov et al., 2015). HmsR and HmsS might be responsible for sensing c-di-GMP and along with the remainder of the *hmsHFRS* operon are responsible for the biosynthesis and translocation of biofilm forming exopolysaccharide (Bobrov et al., 2008; Abu Khweek et al., 2010). Thus, it is the pseudogenization of *rcsA* during the evolution of *Y. pseudotuberculosis* into *Y. pestis* that has allowed the latter to actually form biofilms that enhances flea-borne transmission (Sun et al., 2008; Guo et al., 2015). This likely represents an example of positive Darwinian selection (Zhang, 2008).

### Phage shock proteins and maintenance of proton motive force

Another ECS responsive element is the phage shock protein (Psp) pathway needed for bacterial survival when the inner membrane ion permeability barrier function has been breached (Joly et al., 2010; Yamaguchi and Darwin, 2012). Since proton motive force (PMF) dissolution would follow inner membrane disruption, the Psp pathway's main function is probably to reinstate a PMF to stress-damaged membranes (Jovanovic et al., 2006; Kobayashi et al., 2007; Figure 4D). Hence the efficiency of *sec-*dependent and *tat*-dependent protein secretion across the inner membrane is reliant on a stress-responsive Psp system (Jones et al., 2003; Delisa et al., 2004). A major component of the Psp system is the dynamic PspA protein encoded on the *pspABCDE* operon that is controlled by the PspF transcription factor. In unstressed bacteria, PspA acts as an anti-activator by sequestering PspF. Following stress exposure, PspA is targeted to the membrane to release free PspF to activate *pspABCDE* expression. At the inner membrane, stress alleviation is performed by PspA either alone or together with the integral inner membrane components PspB and PspC. For example, a chief inducer of the Psp response is the mislocalization of outer membrane secretin proteins, while the PspB and PspC proteins specifically work to prevent wrongful secretin insertion into the membrane (Lloyd et al., 2004; Guilvout et al., 2006; Seo et al., 2007; Figure 4D). Given that secretins form outer-membrane channels for the movement of macromolecules across the outer membranes of Gram-negative bacteria (Korotkov et al., 2011; Koo et al., 2012), the Psp response probably supports several aspects of bacterial virulence (Darwin, 2013).

Indeed, *Y. enterocolitica* has served as an excellent model pathogen to study the Psp system in bacterial virulence. An intact Psp system is required for *Y. enterocolitica* survival during active Ysc-Yop T3S when the YscC secretin is naturally over-produced (Darwin and Miller, 2001; Green and Darwin, 2004; Figure 4D). It is believed that this requirement stems from the need for the Psp system to prevent YscC-induced cytolethality upon any mislocalization to the inner membrane during T3S (Horstman and Darwin, 2012). Yet, the Psp system is not actually required for T3SS assembly and function *per se* (Darwin and Miller, 1999), which is a little perplexing considering that the Ysc-Yop T3SS is supposedly reliant on PMF for function (Wilharm et al., 2004). Hence, there is a clear need to better appreciate how the Psp system maintains the PMF in order to understand how energy supplies that drive T3S are preserved (Lee and Rietsch, 2015). It also seems prudent to explore what roles are played by the Psp system in other secretin-dependent protein export and secretion pathways of *Yersinia*.

### Control of protein assembly at the bacterial surface

A prevailing theme during ECS responsiveness is the need to secure protein transport and assembly in and through the bacterial envelope. Quality control of protein folding in the bacterial envelope requires input from dedicated periplasmic protein folding and degradation factors. Hence, the transcriptional regulation of genes encoding these factors are usually wired to the σ^E^ and CpxA-CpxR regulatory networks responsive to ECS, and this ensures their elevated levels at acute times of ECS exposure. Such periplasmic-located quality control factors include: molecular chaperones such as Skp and Spy, folding catalysts such as the disulfide oxidoreductases and the peptidyl-prolyl isomerases (PPIases), and degradosomes such as the DegP/HtrA serine protease (Wick and Egli, 2004; Merdanovic et al., 2011; Mogk et al., 2011; Lyu and Zhao, 2015). While examples of these protein folding and degradation factors can be found in essentially all living organisms, in pathogenic bacteria they are particularly required for production of fully functional virulence determinants that ensure bacterial survival during transit in a host environment.

With this in mind, the contribution of PPIases in *Yersinia* pathogenicity was explored. PPIases are protein chaperones and folding factors that can catalyze proline isomerization in proteins (Gothel and Marahiel, 1999; Fanghanel and Fischer, 2004). Five periplasmic PPIases in *Y. pseudotuberculosis*: SurA, PpiD, PpiA, FkpA, and FklB, have recently been described (Obi et al., 2011), but they are essentially ubiquitous being widespread in other organisms. SurA is implicated in most phenotypic characteristics associated with this protein family (Behrens-Kneip, 2010). In *Yersinia* lacking the *surA* allele, outer membrane perturbations result that include aberrant cellular morphology, drastic alterations in OMP profile, susceptibility to detergents and antibiotics, altered fatty acid and phospholipid composition and leakiness of LPS into the extracellular environment (Obi et al., 2011; Southern et al., 2015). Not surprisingly therefore, the SurA PPIase and chaperone is essential for the virulence of *Yersinia* in a mouse infection models (Obi et al., 2011; Southern et al., 2015). Critically though, SurA^+^ *Yersinia* are also avirulent if they lack all four remaining periplasmic PPIases (i.e., PpiA, PpiD, FkpA, and FklB) (Obi et al., 2011). This suggests that SurA-dependent and SurA-independent pathways are responsible for trafficking essential virulence factors to the *Yersinia* surface. Consistent with this, recent studies have defined possible functions for the PPIases other than SurA in the bacterial cell envelope where under certain conditions they assume important SurA-independent functions in chaperoning, folding and assembly of outer surface proteins once they emerge from the Sec translocon (Antonoaea et al., 2008; Matern et al., 2010; Ge et al., 2014; Gotzke et al., 2014; Sachelaru et al., 2014). Significantly, in *Y. pseudotuberculosis* the PPIases are known to be necessary for proper outer membrane assembly of invasin and Ail, important *Yersinia* adhesins involved in attachment to eukaryotic cells (Obi and Francis, 2013). This is interesting for enteropathogenic *Yersinia* are famed for possessing an abundance of adhesins and secretion systems that are presumed to be important for host cell interactions, but the roles of several of these have not been experimentally proven (Francis, 2011; Mikula et al., 2013). Thus, proteomics approaches can be easily applied to *Yersinia* strains where SurA has been deleted and where PpiA, PpiD, FkpA, and FklB have all been deleted, with the goal to identify novel surface proteins that are essential for survival and/or virulence of *Yersinia*, and also describe the essential proteins in the trafficking and assembly pathways for these surface proteins. Both the surface protein and the trafficking pathway would be candidate targets for anti-infective drug development.

## Metal homeostasis

Decisive for the survival of all organisms is an adequate supply of intracellular transition metals. Minor concentrations of iron, zinc, copper, and manganese are indispensable for countless cellular functions, but when in excess can lead to severe toxicity through disruption of cellular redox potential and production of deleterious reactive hydroxyl radicals that have in turn forced the cell to evolve resistance and detoxification strategies as a safeguard under certain environmental conditions (Hobman and Crossman, 2015; Imlay, 2015). Supply of these transition metals is influenced by oxygen levels and for bacterial pathogens in particular, metal availability is further restricted by the host's innate immune defense (Cassat and Skaar, 2013). In prokaryotes, examples from all major transporter families are known to contribute to metal homeostasis (Klein and Lewinson, 2011). In pathogenic *Yersinia*, most research has focused on mechanisms of iron acquisition, but this does not off-set the importance of other metal acquisition systems given how several of these are up-regulated during *in vivo* growth (Lathem et al., 2005; Sebbane et al., 2006; Table 2).

**Table 2:** **Prominent iron transport systems and associated regulatory factors in pathogenic *Yersinia***.

**System**	**Functional property^a^**
TonB-ExbB-ExbD	Energizer complex
Fur & RyhB	Iron homeostasis in response to iron
ArcA-ArcB & Fnr	Iron homeostasis in response to oxygen
**FERRIC TRANSPORTERS**	
Ybt	Yersiniabactin siderophore system
Ynp	Pseudochelin siderophore system
Ysu	Yersiniachelin siderophore system
Iuc	Aerobactin siderophore system
**FERROUS TRANSPORTERS**	
Yfe	ABC importer of iron and manganese
Feo	Non-ABC-importer
**HEME TRANSPORTERS**	
Hmu	ABC importer
Has	Hemophore system

a *Whether these systems are contained in all three human pathogenic Yersinia has been addressed in part by the study by Forman et al. (2010). This study revealed significant variation in coding potential among the three species and even among strains of a species. Hence, mechanisms of iron transport can have redundant functions or operate only in a specific niche*.

### Iron transport systems in pathogenic *Yersinia*

In facultative anaerobic bacteria such as *Yersinia*, regulation of iron metabolism is linked closely to iron availability and to the levels of oxygen in the environment (Carpenter and Payne, 2014). In anoxic conditions, iron is present in a soluble ferrous (Fe^2+^) form, while in the presence of oxygen, is more commonly found in the insoluble ferric (Fe^3+^) form. Reflecting the importance of iron as a nutrient, bacteria including *Yersinia* have devised an impressive array of iron uptake mechanisms that enable utilization of the two redox forms.

In the presence of oxygen, highly virulent forms of pathogenic *Yersinia* all produce a high pathogenicity island (HPI) encoded yersiniabactin (Ybt) siderophore-based system for the acquisition of Fe^3+^ iron (Forman et al., 2010; Rakin et al., 2012; Table 2). Yersiniabactin production is considered to be an essential virulence determinant for both plague-causing *Y*. *pestis* and also the enteric *Yersinia*, and fitness profiling identifies it as a necessary requirement for optimal growth *in vivo* (Palace et al., 2014). Yet the fact that the *ybt* operon is truncated or even deleted in some strains of virulent *Y. pestis* and *Y. pseudotuberculosis* indicates other modes of Fe^3+^ uptake have evolved. Indeed, this may involve at least three possible alternative siderophore systems–the pseudochelin (Ynp) system, the yersiniachelin (Ysu) system, and the aerobactin (Iuc) system (Forman et al., 2010; Rakin et al., 2012) (Table 2). These particular siderophore-iron complexes are transported back into the cell via coupling to either cognate or generic ABC importers that all consist of a typical ATP binding protein, a periplasmic binding protein, and an outer membrane receptor (Forman et al., 2010) (Table 2). Transport is energized by the universal TonB/ExbBD energizer system and improved iron solubility requires ferric reductase activity. The general importance of the TonB/ExbBD energizer system is reflected in it being selected for optimal *Yersinia* fitness *in vivo* (Palace et al., 2014).

In oxygen limiting conditions, or when reducing agents are present, systems for the transport of Fe^2+^ iron are required. The Yfe and Feo systems appear to be the two predominant ferrous transporters utilized in *Yersinia*, and their functions share some partial redundancy (Perry et al., 2007; Table 2). The Yfe system is a typical ABC importer, which in some bacteria has demonstrated affinity for manganese transport. The Feo system is a non-ABC transporter, which is energized through GTP hydrolysis. Both systems are required for full *Yersinia* virulence in certain infection models (Fetherston et al., 2012). Additionally, when in a mammalian host environment, the potential for pathogenic *Yersinia* to utilize heme as an iron source is made possible via two transport systems—the Hmu ABC transporter and the Has hemophore system (Hornung et al., 1996; Perry and Fetherston, 2004; Forman et al., 2010; Table 2). However, *in vivo* studies suggest that the relative contributions of these two systems to pathogenicity is minimal (Rossi et al., 2001; Forman et al., 2010). Despite this, it seems likely that *Yersinia* have evolved multiple iron transport pathways to ensure their needs for iron are satisfied no matter the prevailing environment, although some of these may make relatively minor contributions to *Yersinia* pathophysiology, at least under the environmental conditions experimentally tested.

### Regulating iron transport systems in pathogenic *Yersinia*

It is not surprising that the synthesis of iron transport systems is tightly controlled in response to available iron and oxygen levels. Responsiveness to iron is through the transcriptional repressor, ferric uptake regulator (Fur), a ubiquitous regulator in all prokaryotes (Troxell and Hassan, 2013; Fillat, 2014; Table 2). In fact, metal homeostasis is generally achieved by a large superfamily of Fur-like proteins that also specifically regulate the genes of other transition metal uptake systems (Fillat, 2014). In iron replete conditions, Fur binds Fe^2+^ to form homodimers that bind to DNA target sequences in iron-responsive gene promoters that represses their transcription through RNA polymerase occlusion. When concentrations of iron are low, Fe^2+^ is displaced from Fur, which now assumes a monomeric state that is unable to interact with DNA, allowing transcription from iron-responsive promoters to proceed. The iron-Fur regulon in *Y. pestis* has been studied by DNA microarray, biochemical and *in silico* analyses. Predictably, Fur and high iron concentrations repressed a number of operons that reinforced their involvement in iron homeostasis (Zhou et al., 2006; Gao et al., 2008). However, as in other bacteria the Fur protein was also observed to activate the transcription of a few genes (Zhou et al., 2006). It is assumed that this action occurs through the small non-coding RNA, RyhB (see below; Table 2). RyhB is a post-transcriptional repressor of gene expression and its expression is repressed by Fur (Salvail and Masse, 2012). Thus, when Fur is inactive at low iron concentrations, RyhB is generated to specifically promote the degradation of mRNA's that encode for a number of non-essential iron utilization genes that consequently liberates free iron for essential purposes (Salvail and Masse, 2012). In *Y. pestis*, the function of RyhB has been studied to some extent. Two Fur-regulated RyhB homologs are expressed, and while both are up-regulated during *in vivo* infections in a mouse model (Deng et al., 2012; Yan et al., 2013), neither of the two were essential for virulence under the inoculation routes tested (Deng et al., 2012).

Iron transport is also regulated in response to oxygen, which is necessary so that bacteria can adapt transport systems to the different forms of iron (Carpenter and Payne, 2014). Adaptation can occur by the direct action of global regulators of respiration at transport gene promoters or indirect through changes in Fur levels. In the latter, *fur* expression is repressed under iron-replete conditions through a feedback auto-regulatory loop, and in several systems is induced by the redox regulator OxyR in response to oxidative stress (Troxell and Hassan, 2013; Carpenter and Payne, 2014; Fillat, 2014). An increase in Fur levels in oxidative stress conditions will reduce iron uptake and subsequently the risk of hydroxyl radical formation by the Fenton reaction, but also lead to the upregulation of key strategies for the detoxification of these radicals (Troxell and Hassan, 2013; Carpenter and Payne, 2014; Fillat, 2014). Nevertheless, evidence for this Fur-OxyR regulatory pathway in *Yersinia* is still lacking (Fetherston et al., 2012).

Global regulators of respiration include the Fnr oxygen sensitive transcription factor and ArcA that is part of the ArcA-ArcB TCRS (Table 2). Fnr is a major global transcription factor, and uses self-contained iron-sulfur clusters to sense prevailing oxygen levels (Korner et al., 2003; Green et al., 2009). Homodimerization occurs in anaerobic conditions and these homodimers bind DNA to completely reprogram the cell from aerobic to anaerobic respiration. ArcA activation is linked to the ability of ArcB to sense redox potential and electron transfer among the soluble membrane electron carriers under anoxic and oxic conditions (Malpica et al., 2006). Among bacteria of the Enterobacteriaceae, both active Fnr and ArcA regulate the expression of genes belonging to iron transport systems using both Fur-dependent and—independent mechanisms. However, a whole genome promoter structure analysis to identify regulation during anaerobic respiration failed to predict these mechanisms in either the plague or enteric *Yersinia* (Ravcheev et al., 2007), and this is also supported by limited experimentation (Fetherston et al., 2012). Nevertheless, independent studies highlight the importance of both Fnr and Arc systems in the adaptation of *Yersinia* to *in vivo* growth environments (Palace et al., 2014; Avican et al., 2015), which makes them likely to be a cornerstone for metabolic adaptation during pathogenesis.

### Other (non-iron) metal acquisition systems in pathogenic *Yersinia*

Due to their roles as cofactors in metal-containing proteins, many additional non-iron metals are also essential for cell function. Of these, zinc, manganese, copper, and nickel transporters are among the better characterized non-iron homeostasis systems in the Enterobacteriaceae (Porcheron et al., 2013). Higher organisms often limit the availability of these essential trace metals to lessen the threat of pathogen infection, in a process termed “nutritional immunity” (Becker and Skaar, 2014). As a counteractive measure, bacterial pathogens encode for host-inducible influx systems that are designed to sequester limiting trace metals from the host (Lathem et al., 2005; Sebbane et al., 2006). Although not studied to the same extent as iron scavenging systems, transporters for zinc and manganese are now established as an integral part of the *Yersinia* physiological makeup (Porcheron et al., 2013; Perry et al., 2015).

To date, there exists three known modes of zinc acquisition in *Yersinia*. The first is the ZnuABC importer common among many prokaryotes (Desrosiers et al., 2010). The second is the iron siderophore, yersiniabactin (Ybt; Bobrov et al., 2014), and the third is YezP, a zinc binding protein surprisingly secreted by one of the type 6 secretion systems of *Y. pseudotuberculosis*, designated T6SS4 (Wang et al., 2015). Despite this knowledge, the actually manner in which Zn^2+^ crosses the outer membrane is not known for any of these three systems. Moreover, it is not known how the Zn^2+^-laden Ybt and YezP then moves across the inner membrane for entry back into the *Yersinia* cytoplasm, although the utilization of Zn^2+^-Ybt is dependent on the inner membrane permease YbtX (Bobrov et al., 2014). As these two systems are unusual, this suggests that truly novel uptake mechanisms are waiting to be uncovered. As alluded to already in this section, zinc homeostasis operons in *Yersinia* are under the typical control of Zur, a zinc-responsive transcriptional repressor belonging to the Fur-like superfamily (Li et al., 2009).

Manganese in the form of Mn^2+^ is preferentially used by all biological systems. So far, two Mn^2+^ importers have been characterized in pathogenic *Yersinia*. The first is a rather common proton-dependent symporter termed MntH (Champion et al., 2011; Perry et al., 2012), an ortholog to the eukaryotic Nramp1 symporter that assists in the elimination of pathogens by restricting their access to divalent cations in the phagosome (Cellier et al., 2007). It follows that MntH enables intracellular *Y. pseudotuberculosis* to accumulate manganese and to resist antimicrobial killing by phagocytes producing Nramp1 (Champion et al., 2011). In *Y. pestis*, manganese transport could well be niche specific for bacteria lacking *mntH* were attenuated in a bubonic, but not pneumonic, mouse model of infection (Perry et al., 2012). The second influx system is the ABC transporter YfeABCD, which has dual specificity for both iron and manganese (Bearden and Perry, 1999). In some organisms, manganese homeostasis is controlled by a Fur-like transcriptional repressor termed Mur (Fillat, 2014), although the most common form of control in the Enterobacteriaceae involves the MntR regulator (Porcheron et al., 2013). Yet *Y. pestis* has evolved without the Mur or MntR regulators (Perry et al., 2015), so the manganese transporters Yfe and MntH are actually Fur-regulated (Perry et al., 2012).

## Metabolism and virulence

**Figure 5:**
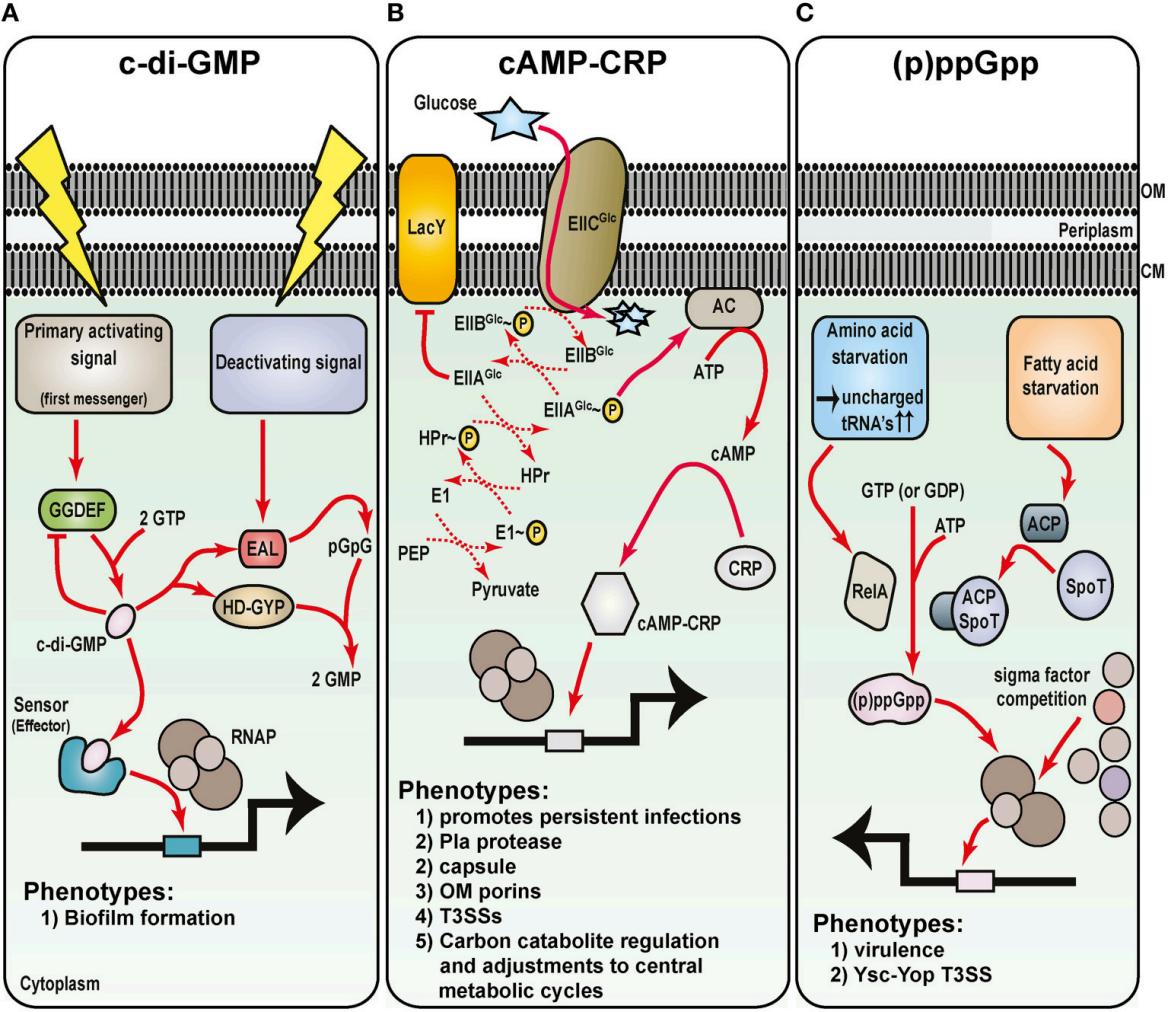
**Metabolic intermediates regulate *Yersinia* virulence**. Understanding the role of nucleotide-based second messengers in the regulation of *Yersinia* survival and virulence is still in its infancy. As determined from many studies in *E. coli* systems, three major regulatory molecules are known: **(A)** c-di-GMP, **(B)** cAMP and its receptor Crp, and **(C)** ppGpp and pppGpp [collectively known as (p)ppGpp]. Primary activation signals control diguanylate cyclase (GGDEF domain containing proteins) to generate c-di-GMP **(A)**. Various effector molecules then interact with c-di-GMP to regulate gene expression, including Hms-mediated biofilm formation. Deactivating signals stimulate phosphodiesterase activity of proteins containing either EAL or HD-GYP domains, and this degradation pathway ensures that c-di-GMP levels are stringently controlled. Glucose availability and the phosphoenolpyruvate (PEP)—carbohydrate phosphotransferase system (PTS) control cAMP production by adenylate cyclase (AC) **(B)**. Upon cAMP production when glucose is limiting, cAMP-CRP complexes form and this enhances RNAP holoenzyme binding to vast numbers of sensitive promoters including several secreted *Yersinia* virulence factors. Finally, various forms of starvation stimulates RelA-dependent and SpoT/acyl carrier protein (ACP)-dependent synthesis of (p)ppGpp **(C)**. By direct binding to the RNA polymerase, (p)ppGpp can influence sigma factor competition for core RNAP, and through this affects transcription of a plethora of genes that in *Yersinia* includes the prominent Ysc-Yop plasmid-encoded T3SS.

It has long been appreciated that bacterial pathogens produce an array of virulence factors to counteract the antibacterial immune responses from the host, and in turn this creates a favorable niche for bacterial colonization and survival. However, underappreciated until relatively recently is the need for bacteria to seek the necessary nutrients required to maintain energy levels to sustain bacterial replication in a host environment. Although, eukaryotic cells or tissues can provide an extensive source of nutrients, the variety and degree of available nutrients is often both spatially and temporally restricted. Hence, as bacteria transit through different host compartments and tissues, they must rapidly adjust their central metabolism to make efficient use of what nutrients are available. Interestingly, new evidence is emerging to indicate that pathogens have streamlined adaptive processes to couple metabolic activity to a defined program of virulence gene expression (Eisenreich et al., 2010; Rohmer et al., 2011; Heroven and Dersch, 2014; Wilharm and Heider, 2014). To some extent, this has been long forecast by the knowledge that common nucleotide metabolic derivatives—cyclic AMP, cyclic di-GMP, and the guanosine polyphosphate molecules ppGpp and pppGpp (Figure 5) play profound roles as signaling molecules in the control of bacterial gene expression (Kalia et al., 2013).

### c-di-GMP signaling network in *Yersinia* biofilm formation

Cyclic dimeric guanosine monophosphate (c-di-GMP; cyclic diguanylate) is a ubiquitous nucleotide-second messenger in bacteria that regulates many physiological process. Most often this culminates in transitioning between a single-cell planktonic lifestyle to a multicellular sessile lifestyle in biofilm communities (Romling et al., 2013). Impacted by various environmental cues, such as oxygen, nitric oxide, light, nutrients, and temperature (Kalia et al., 2013), c-di-GMP is synthesized from two GTP molecules by diguanylate cyclases containing GGDEF domains (Figure 5A). Specific phosphodiesterases associated with EAL or HD-GYP domains are responsible for c-di-GMP degradation (Romling et al., 2013; Figure 5A). The biochemistry of c-di-GMP synthesis and degradation is quite obviously complex. This is brought about by the fact that a bacterial genome encodes for quite a few different proteins that harbor either a GGDEF or EAL domain, and there exits even examples of both domains being present in the same protein. Moreover, c-di-GMP is bound by diverse receptors that for example, includes PilZ-domain containing proteins and riboswitches (Romling et al., 2013). These findings have important functional consequences. Clearly, c-di-GMP levels must be in constant flux, and this creates critical concentration gradients in various foci throughout the cytoplasm. Hence, at a particular threshold concentration in a given location within the cytoplasm, c-di-GMP is capable of exerting its regulatory activity at multiple all levels ranging from transcription to post-translation.

Bioinformatics prediction suggests as many as 8 GGDEF and/or EAL domain-containing proteins encoded by the *Y. pestis* genome (Darby, 2008). In the *Y. enterocolitica* genome, this number swells to a possible 22 proteins (Heermann and Fuchs, 2008). These observations point to a complex c-di-GMP-mediated regulatory network in the control of biofilm formation at ambient temperature in pathogenic *Yersinia* (Zhou and Yang, 2011). In addition to the major *hmsHFRS* locus responsible for EPS synthesis and transport, *Y. pestis* harbors the *hmsT, hmsCDE*, and *hmsP* operons that encode for regulatory components. Among them are the two digualylate cyclases HmsT and HmsD responsible for c-di-GMP synthesis, and the HmsP phosphodiesterase responsible for c-di-GMP turnover (Jones et al., 1999; Kirillina et al., 2004; Bobrov et al., 2005, 2011; Simm et al., 2005; Sun et al., 2011; Ren et al., 2014). In addition, HmsB, a temperature dependent small non-coding regulatory RNA stimulates c-di-GMP levels by enhancing the expression of *hmsB, hmsT, hmsCDE*, and *hmsHFRS*, and repressing *hmsP* (Fang et al., 2014; Figure 5A). Consistent with HmsB involvement is the observation that Hfq, the premier RNA chaperone (see later), is also described to influence *Yersinia* biofilm formation, although some disparity exists between the two studies concerning the manner in which Hfq is thought to exert its affect (Bellows et al., 2012; Rempe et al., 2012). Nevertheless, it is still very evident that *Y. pestis* biofilms form following a build-up of c-di-GMP, but are repressed by the metabolism of c-di-GMP. In fact, the ability to accumulate c-di-GMP through incremental gene loss was a dominant force driving the evolution of *Y. pestis*, for it enabled biofilm formation in the flea foregut, which enhanced transmissibility from the flea vector (Sun et al., 2014).

The receptors of c-di-GMP are not yet experimentally defined. Hence, it is not really clear how direct c-di-GMP signaling is in regulating *Y. pestis* biofilm formation. Indeed, multiple factors controlling *Yersinia* biofilm formation are known, so the regulatory network is extremely complex. As mentioned earlier in this review, the Rcs phosphorelay system directly regulates *Yersinia* biofilm formation through repression of various *hms* loci (Hinchliffe et al., 2008; Sun et al., 2012; Fang et al., 2015; Guo et al., 2015). The classical PhoQ-PhoP two component regulatory system is also required for controlled biofilm formation (Sun et al., 2009b; Rebeil et al., 2013), as is the newly described LysR-type transcriptional regulator, y*fbA* (Tam et al., 2014). Additionally, deficiency of the polyamine putrescine restricts biofilm formation due to post-transcriptional defects in the synthesis of key Hms components that are required for biosynthesis of EPS (Patel et al., 2006; Wortham et al., 2010). Moreover, the NghA glycosyl hydrolase reduces biofilm formation by degrading extracellular matrix constituents (Erickson et al., 2008). Finally, a role for quorum sensing in enhancing biofilm formation by enteropathogenic *Yersinia* has been reported, but this appears indirect through quorum sensing-dependent repression of type III secretion (Atkinson et al., 2011). Yet, with all the varied positive and negative regulators of *Yersinia* biofilm formation described, the real challenge now is to integrate these into a cohesive and dynamic regulatory network.

### Carbon catabolite repression—cAMP-CRP

The Enterobacteriaceae utilize glucose as the preferred carbon source. Glucose limiting environments promote cross-talk between the phosphoenolpyruvate-carbohydrate phosphotransferase system, adenylate cyclase (producer of the second messenger cyclic AMP—cAMP), and CRP (cAMP receptor protein; Deutscher, 2008; Gorke and Stulke, 2008; Figure 5B). This cross-talk specifically reprograms bacteria to activate secondary carbon scavenging and utilization pathways to maintain growth. Referred to as carbon catabolite repression, this regulatory process enables bacterial to preferentially use glucose prior to the metabolism of other non-preferred carbon nutrients (Deutscher, 2008; Gorke and Stulke, 2008). The regulatory mechanism relies upon enhanced production of cAMP by adenylate cyclase when glucose is limiting. When cAMP is bound by CRP, a global transcription activator is formed that is competent to bind DNA promoter sequences to reprogram gene expression so that bacteria can make use of new carbon nutrient sources (Lawson et al., 2004; Won et al., 2009).

It is quite common for cAMP-Crp dependent reprograming of gene expression to include alterations in virulence gene expression. In fact, the cAMP-Crp regulatory system of *Yersinia* is a vital player for pathogenicity (Zhan et al., 2008; Heroven et al., 2012b; Lathem et al., 2014; Avican et al., 2015), and this includes specific control of the Pla protease, components of T3SSs, the F1 capsule, outer membrane porins, and virulence associate carbon storage regulatory (Csr) system small non-coding RNAs (Petersen and Young, 2002; Kim et al., 2007; Zhan et al., 2008, 2009; Gao et al., 2011; Heroven et al., 2012b) as well as non-specific control over biofilm formation (Willias et al., 2015; Figure 5B). However, recent work has begun to shed light on the shear scope of cAMP-Crp involvement in *Yersinia* pathophysiology. Working in concert with the Csr system and the major transcriptional regulator RovA (Heroven et al., 2008, 2012a,b), this Crp-Csr-RovA regulatory cascade functions to control the sophisticated carbon metabolism network at the level of the pyruvate- tricarboxylic acid cycle, and these core metabolic adjustments are necessary for *Yersinia* to successfully transition from free-living to infectious state (Bucker et al., 2014). This process includes modulating virulence gene expression so that *Yersinia* can switch from acute infection to chronic persistent infection in an *in vivo* mouse model (Avican et al., 2015; Figure 5B). Reflecting this transition from free-living to infectious state, temperature up-shift from 26 to 37°C results in a dramatic reprogramming of the cAMP-Crp regulon (Nuss et al., 2015) and an adjustment of catabolic pathways in readiness to utilize nutrients derived from the host (Motin et al., 2004; Chromy et al., 2005). Several small non-coding regulatory RNAs are among the vast cAMP-Crp regulon, which suggests coupling between metabolism and virulence occurs at the post-transcriptional level (Nuss et al., 2015). This idea is supported by a multi-omics systems approach that revealed mechanisms of post-transcriptional control of metabolism that are conserved between *Y. pestis* and *Y. pseudotuberculosis* (Ansong et al., 2013).

It is obvious that the regulation of *crp* gene expression is then a significant process in *Yersinia*. In *Y. pestis*, the PhoQ-PhoP system directly regulates *crp* expression at the transcriptional level (Qu et al., 2013; Zhang et al., 2013b), whereas in *Y. pseudotuberculosis* it is the *csr* system that is transcriptionally regulated by PhoP (Nuss et al., 2014). Recently, a mechanism of positive post-transcriptional regulation of Crp production was found to involve the 5′ untranslated region of *crp* mRNA, temperature and the RNA binding protein Hfq (Lathem et al., 2014). This finding goes a long way to explaining the contributions of Hfq to the metabolic fitness and virulence of pathogenic *Yersinia* (Geng et al., 2009; Bai et al., 2010; Schiano et al., 2010; Bellows et al., 2012; Kakoschke et al., 2014). Further investigation of *crp* regulation will benefit understanding of how *Yersinia* adapts metabolic and virulence capacity during host infections.

### Stringent response and (p)ppGpp

The bacterial stringent response is starvation induced and leads to accumulation of guanosine tetraphosphate (ppGpp) and guanosine pentaphosphate (pppGpp). Commonly referred to as (p)ppGpp, these second messengers coordinate multiple physiological processes within the bacterial cell in response to nutrient and environmental stress (Gaca et al., 2015; Hauryliuk et al., 2015). Regulation primarily occurs at the transcriptional level through alterations of promoter selection by RNA polymerase, but can also occur at the post-transcriptional level (Dalebroux and Swanson, 2012). The RelA-SpoT homolog (RSH) family enzymes are responsible for synthesis and hydrolysis of (p)ppGpp (Figure 5C). The RelA-SpoT pathway functions in β- and γ-proteobacteria, whereas a Rel pathway is much more widely distributed among prokaryotes (Gaca et al., 2015; Hauryliuk et al., 2015). At least in the Enterbacteriaceae, the RelA pathway is responsive to limiting amino acids and uncharged tRNA accumulation. The SpoT pathway collaborates with acyl carrier protein and is responsive to limiting phosphate, carbon, or fatty acids. Not surprisingly, (p)ppGpp is an active player in the regulation of virulence gene expression (Dalebroux et al., 2010). Although not well-studied in pathogenic *Yersinia*, a *Y. pestis relA, spoT* double mutant cannot accumulate (p)ppGpp and is attenuated in virulence (Sun et al., 2009a). One reason for this attenuation could well be an inactive Ysc-Yop T3SS (Sun et al., 2009a). Given the importance of this T3SS to *Yersinia* virulence, follow-up studies to understand how (p)ppGpp impacts on its activity would be beneficial.

### New concepts in metabolism and virulence circuitry

With new technologies advancing knowledge of bacterial metabolism *in vivo*, it is now apparent that novel bacterial factors can be deployed to profoundly affect the metabolic status of the host which in turn augments bacterial virulence (Abu Kwaik and Bumann, 2013; Staib and Fuchs, 2014). A seminal paper in 2009, revealed a distinct physical cross-talk between metabolism and the production of type III secretion by *Yersinia* (Schmid et al., 2009). In particular, this study demonstrated that virulence plasmid-encoded Ysc-Yop T3SS synthesis and activity was inversely correlated to the levels of available oxaloacetate derived amino acids, via direct T3SS-dependent control of phosphoenol pyruvate carboxylase activity. This study served to garner the then fleeting concept that the activity of classical virulence determinants was purposefully linked to the control of nutrients essential for niche-specific *in vivo* growth.

Now with the routine use of systems level-based applications in infection biology research, the identification of metabolic factors with direct coupling to virulence is increasing steadily. For example, transcriptomics data from several studies designed to assess how *Yersinia* adapts to a mammalian host environment revealed an obvious and substantial commitment to reprogram carbon uptake and utilization strategies for the purpose of optimizing *in vivo* growth to suit prevailing nutritional availability (Lathem et al., 2005; Rosso et al., 2008; Fukuto et al., 2010; Ansong et al., 2013; Pradel et al., 2014), and these observations corroborated independent *in vivo* fitness profiling data of bacteria deficient in core metabolic pathways (Palace et al., 2014; Pradel et al., 2014; Avican et al., 2015; Deng et al., 2015). Within this context, Sasikaran and colleagues have recently demonstrated that *Y. pestis* and *Pseudomonas aeruginosa* possessed the capacity to metabolize itaconate, a mammalian metabolite with potent anti-bacterial properties (Sasikaran et al., 2014). This is significant because possession of functional itaconate degradation genes in *Y. pestis* is required for full pathogenicity (Pujol et al., 2005). The reality is that control of carbon utilization pathways to exploit prevailing environmental niches is believed to be a common phenomenon in diverse bacteria as they transition through a host. It follows that a new paradigm “nutritional virulence” has begun to take hold, and is designed to encapsulate the importance to pathogenicity of pathogen-directed exploitation of host nutrients (Abu Kwaik and Bumann, 2013). Hence, an increased understanding of bacterial metabolic mechanisms supporting optimal *in vivo* growth may eventually lead to novel strategies for the treatment and prevention of bacterial diseases. With biologically relevant acute and chronic *in vivo* infection models in place, studies designed to interrogate *Yersinia* pathophysiology are well-placed to shed further light on this nutritional virulence concept.

## Post-transcriptional control by small non-coding regulatory RNA's

Small regulatory RNAs have a tremendous impact upon physiological and metabolic circuits in bacterial model organisms, such as *E. coli* K12 and *Bacillus subtilis*. The overall impact of small regulatory RNAs (sRNAs) on the pathogenesis and environmental adaptation of bacteria of medical importance cannot be understated. While investigations into *Yersinia* sRNAs are not completely novel, their magnitude and frequency are increasing. The initial foray into global sRNA biology in *Yersinia* species began with bioinformatics searches for homologs of small RNAs characterized in *E. coli* (Delihas, 2003). In general, small RNAs can be categorized by their dependence upon the RNA-chaperone Hfq. In fact, Hfq is a co-factor for the largest class of sRNAs in *E. coli*. These sRNAs require Hfq for their activity, as they bind to Hfq and facilitate interaction with mRNA targets. The Hfq-dependent small regulatory RNAs are trans-acting and act by complementary base pairing to a distal mRNA target. The Hfq-independent small RNAs act as *cis*-antisense RNAs or protein-binding RNAs.

Interestingly, Nakao and colleagues reported already in 1995 that the *Y. enterocolitica* heat-stable toxin (Y-ST) required the *E. coli* Hfq homolog for maximal production (Nakao et al., 1995); an observation suggesting that *Y. enterocolitica* heat-stable toxin (Y-ST) may be regulated by an as yet unknown sRNA. In fact, it is now known that Hfq is required for full virulence of all three pathogenic *Yersinia* species (Geng et al., 2009; Schiano et al., 2010; Kakoschke et al., 2014), and this reflects clearly on the importance of sRNA's and numerous post-transcriptional regulatory mechanisms in the control of *Yersinia* pathogenic mechanisms. Consequently, there are now numerous investigations into *Yersinia* small RNA biology. Many of these have identified unique *Yersinia* specific sRNAs that are not homologous to other sRNA sequences within other enteric bacteria. In this section, we will review the literature on both Hfq-dependent, Hfq-independent sRNA, and unique *Yersinia* specific sRNAs (Ysrs). We will also highlight functional characterization that was executed using surrogate genetics in *E. coli* vs. studies within the native *Yersinia* organisms.

### Genome-wide searches for small regulatory RNAs in *Yersinia* sp.

Genome wide searches in *E. coli* led to the discovery of a significant amount of prokaryotic model small RNAs. Once these small RNAs were characterized, significant insights into physiology were uncovered in the areas of outer membrane metabolism, iron metabolism, envelope stress, and carbon metabolism. Inevitably, these genome wide searches were expanded into other bacteria including *Yersinia* species, yielding the identification of sRNAs homologous to many of these small RNAs. Deep sequencing of total RNA libraries from *Y. pseudotuberculosis* resulted in the discovery of approximately 150 previously unannotated sRNAs, defined as *Yersinia* small RNAs or Ysr (Koo et al., 2011). Thirty two of these Ysr's were orthologous to previously characterized sRNAs encoded within the *E. coli* and *S. typhimurium* genomes. The remaining 118 Ysr sequences were unique to specific *Y. pseudotuberculosis* and *Y. pestis* genomes (Koo et al., 2011). Ysr expression was confirmed by Northern Blot analysis and animal studies demonstrated that selected Ysr deletions were attenuated for virulence in a mouse model of infection (Koo et al., 2011).

### Investigations and insights into *Yersinia* small RNA homologs using surrogate genetics in *E. coli*

#### GcvB

*E. coli* GcvB is a 205 nucleotide long sRNA regulated by the Glycine cleavage system regulators GcvA and GcvR. It post-transcriptionally regulates periplasmic transport proteins OppA and DppA. In contrast to the *E. coli gcvB* gene, *Y. pestis* KIM6 *gcvB* encodes for two small RNAs (Table 3). Using surrogate genetics in *E. coli*, McArthur and colleagues demonstrated that expression of *Y. pestis* KIM6 *gcvB* sRNAs leads to repression of *E. coli dppA* expression *in vivo* (McArthur et al., 2006). Furthermore, the expression of *Y. pestis* KIM6 *gcvB* sRNAs are regulated by *Y. pestis* KIM6 *gcvA* and *gcvB* gene products, with GcvA acting as an activator and GcvB acting as a repressor (McArthur et al., 2006). A *Y. pestis* KIM6 *gcvB* mutant has a different growth rate and colony morphology than wild type cells, with *gcvB*- cells appearing dry and compact as opposed to smooth and sticky (McArthur et al., 2006).

**Table 3:** **Investigations of non-*Yersinia* Specific sRNA in different species**.

**Surrogate genetic investigations in *E. coli***	**Investigations in *Yersinia* species**		
	***Y. pseudotuberculosis***	***Y. enterocolitica***	***Y. pestis***
GcvB (*Y.pestis*)	SraG	MicF	RyhB1
SgrS (*Y. pestis*)	CsrB		RyhB2
GlmY (*Y. pseudotuberculosis*)	CsrC		CyaR
GlmZ (*Y. pseudotuberculosis*)			SsrA

#### SgrS

*E. coli* SgrS is a sRNA that modulates sugar-phosphate stress (Vanderpool and Gottesman, 2007). *Y. pestis sgrS* homologs were functionally analyzed using a surrogate genetics approach (Wadler and Vanderpool, 2009; Table 3). It was demonstrated that the over-expression of *Y. pestis* SgrS in *E. coli* Δ*sgrS* mutants resulted in *sgrS* complementation (Wadler and Vanderpool, 2009). Specifically, *Y. pestis* SgrS expression resulted in repression of *ptsG* expression and abrogation of sugar-phosphate toxicity, consistent with the function of *E. coli* SgrS (Wadler and Vanderpool, 2009).

#### GlmY and GlmZ

GlmY and GlmZ are two small regulatory RNAs in *E. coli* involved in the post-transcriptional regulation of *glmS*, encoding the L-glutamine:D-fructose-6-phosphate aminotransferase (GFAT), or Glucosamine-6-phosphate transferase (Urban et al., 2007; Urban and Vogel, 2008). The transcriptional regulation of *Y. pseudotuberculosis glmY* and *glmZ* was characterized using a surrogate genetic approach in *E. coli* (Gopel et al., 2011; Table 3). In this study it was demonstrated that *Y. pseudotuberculosis glmY* and *glmZ* expression are regulated by σ^54^ (σ^N^), GlrR, and IHF suggesting that GlmY and GlmZ are regulated by nitrogen metabolism *in vivo* (Gopel et al., 2011).

### *Yersinia*-specific investigations using genetics and molecular biology

#### SraG

SraG is a 146-174 nt sRNA originally found in a genome wide search for novel sRNAs (Argaman et al., 2001). The SraG homolog in *Y. pseudotuberculosis* YPIII was characterized *in vivo* using genetics and proteomic analyses (Lu et al., 2012; Table 3). A proteomic screen of YPIII identified 16 proteins that were modulated by SraG depletion (Lu et al., 2012). The putative protein targets with the strongest positive modulation include flagellar motor switch protein G (*fliG*–YPK_2392), maltose regulon periplasmic protein (*malM*–YPK_0382), 50S ribosomal protein L9 (*rpll*–YPK_3781), Glutamine ABC transporter (*glnH*–YPK_1600), and an uncharacterized protein likely involved in high-affinity Fe^2+^ transport (YPK_2251; Lu et al., 2012). The putative protein targets with the strongest negative modulation include polynucleotide phosphorylase / polyadenylase (*pnp*–YPK_3726) and protein of unknown function YPK_1205 (Lu et al., 2012). Subsequent analyses confirmed that YPIII SraG sRNA regulates post-transcriptional production of YPK_1205 (Lu et al., 2012). Specifically, mutation in *sraG* resulted in increased YPK_1205 transcript levels as measured by ß-galactosidase activity and over-expression of *sraG* led to decreased YPK_1205 cDNA levels (Lu et al., 2012).

#### MicF

The MicF sRNA was identified and annotated in *Yersinia enterocolitica* by bioinformatics (Delihas, 2003; Table 3). In this study was also identified predicted interactions between *Y. enterocolitica* MicF and OmpF. Experimental analyses of *Yersinia* small RNAs was executed in *Y. pestis*, where the predicted interaction was likely conserved (Liu et al., 2015). Unlike *E. coli, Y. pestis* MicF over-expression resulted in stimulation rather than repression of OmpF. In a strain of *Y. pestis* cured of plasmid pPCP1, MicF repression of OmpF was restored (1.8- vs. 5-fold in *E. coli*), suggesting that stimulation was due to the presence of endogenous plasmid pPCP1 (Liu et al., 2015).

#### RyhB

RyhB is an 80 nt sRNA originally discovered in *E. coli* (Masse and Gottesman, 2002). It is regulated by the Ferric uptake regulator, Fur, and its expression is induced under iron-limited conditions (Masse and Gottesman, 2002). RyhB post-transcriptionally regulates the production of several iron-sulfur cluster proteins in *E. coli* (Masse and Gottesman, 2002; Masse et al., 2003). *E. coli* RyhB has a relatively large regulon and significant impact on the physiology of the cell (Masse and Gottesman, 2002; Masse et al., 2005). RyhB homologs have been discovered in several phylogenetically divergent species, starting with those most closely related to *E. coli*, as RyhB is highly conserved amongst enteric bacteria like many of the Hfq-dependent sRNAs in *E. coli* (Masse and Gottesman, 2002). Comparative genomic analyses led to the identification of two RyhB homologs in *Y. pestis*, RyhB1 and RyhB2 (Deng et al., 2012; Table 3). Both RyhB1 and RyhB2 levels are responsive to iron depletion, the presence of *hfq*, and the presence of *fur* (Deng et al., 2012). However, RyhB2 is less stable in the absence of *hfq* whereas the stability of RyhB1 is not affected by the absence of *hfq* (Deng et al., 2012). Analyses of *Y. pestis* RyhB expression in a mouse model of infection demonstrated a significant induction of RyhB1 and RyhB2 expression in lung tissue compared to spleen and growth *in vitro* in BHI (Deng et al., 2012; Yan et al., 2013). However, neither the *ryhB1* or *ryhB2* mutants demonstrated attenuation for virulence (Deng et al., 2012). The steady-state levels of *Y. pestis* RyhB1 and RyhB2 are modulated by the activity of ribonucleases (Deng et al., 2014). Specifically, RNaseIII is necessary for the accumulation of both RyhB1 and RyhB2 via repression of PNPase levels in the absence of Hfq (Deng et al., 2014). The half-life of RyhB1 and RyhB2 levels were increased in the absence of *pnp*, confirming that PNPase affects stability of *Y. pestis* RyhB1 and RyhB2 (Deng et al., 2014).

### The cAMP receptor protein (Crp) regulatory circuit and small RNAs

The global regulator involved in carbon metabolism, Crp, regulates and is regulated by several small RNAs in *Yersinia* species. In *E. coli* and *Salmonella*, CyaR is a Crp regulated sRNA. CyaR is widely conserved amongst Gram-negative enteric bacteria. The genome wide search excuted by Yan and colleagues, and discussed above, yielded the identification of *Y. pestis* CyaR as well as three small RNAs as new members of the *Y. pestis* Crp regulon (Yan et al., 2013; Table 3). Those three small RNAs, sR065, sR066, and sR084, are encoded within intergenic regions of the pPCP1 plasmid (Yan et al., 2013). Primer extension, northern blot analyses, and computational analyses suggested that sR084 is positively regulated by Crp and sR066 is negatively regulated by Crp (Yan et al., 2013).

Hfq was picked up in a screen to identify factors that regulate *Y. pestis* Pla activity (Plasminogen activator; Lathem et al., 2014). In this study it was demonstrated that Hfq regulated Pla synthesis post-transcriptionallly in a manner independent of the *pla* 5′ UTR. This led to the hypothesis that *Y. pestis* Hfq post-transcriptionally regulated *crp* transcript levels. Post-transcriptional expression of *crp* was decreased in the absence of *hfq* (Lathem et al., 2014). The authors further demonstrated that multi-copy *crp* expression leads to induction of *pla* and partial suppression of the Δ*hfq* growth defect (Lathem et al., 2014).

RNA sequencing analyses of *Y. pestis* grown *in vitro* and in an animal model of infection led to the identification of over 10 Crp-regulated sRNAs (Nuss et al., 2015). Three of these were novel sRNAs, not identified in previous screens (Nuss et al., 2015). The Crp-dependent regulation of these sRNAs were temperature variable, with the vast majority of the regulation observed at 25 vs. 37°C (Nuss et al., 2015). The authors identified 1 novel *trans*-encoded RNA (Ysr206) and two *cis*-antisense RNAs (Ysr232 and Ysr114) (Nuss et al., 2015). Crp also regulates the Hfq-independent sRNAs CsrB and CsrC, and these RNAs are discussed further down.

### Yop-Ysc Type III secretion and small regulatory RNAs

In *E. coli* and other bacteria, the SmpB (small protein B)–SsrA (small stable RNA A) system is used for protein quality control. SsrA (or tmRNA) is a very unique RNA molecule that relieves ribosome stalling through its combined action as both an mRNA and a tRNA. SsrA enters stalled ribosomes as an alanine-charged tRNA mimic, the stalled polypeptide chain is then transferred to the alanine charged SsrA molecule via transpeptidation (Roche and Sauer, 1999). SsrA then enters the ribosome as an mRNA molecule and is translated resulting in the addition of an AANDENYALAA peptide tag to the stalled polypeptide. C-terminally AANDENYALAA tagged proteins are marked for degradation by the ATP-dependent protease ClpXP (Keiler et al., 1996; Gottesman et al., 1998). SmpB binds to SsrA and acts as a cofactor for SsrA, assisting its SsrA binding to ribosomes and ClpXP (Karzai et al., 1999; Wah et al., 2002). In contrast to the post-transcriptional regulators of RNA stability or translational initiation exhibited by the vast majority of small regulatory RNAs, SsrA is unique amongst small regulatory RNAs and was identified prior to the contemporary genome wide screens were executed. The SmpB-SsrA system is active and contributes to the physiology of *Y. pseudotuberculosis* (Okan et al., 2006; Table 3). Specifically, *Y. pseudotuberculosis* deleted for SmpB and SsrA (designated ΔBA) is attenuated for virulence in a mouse model of infection and is defective for proliferation in macrophages (Okan et al., 2006). The SmpB-SsrA locus is also necessary for the secretion of Yops (Okan et al., 2006). Specifically, the ΔBA mutant exhibited decreased mRNA and protein levels of the secreted substrates YopB, YopD, YopE, and LcrV (Okan et al., 2006).

The identification of Hfq in the regulation of T3SS in *Y. pseudotuberculosis* (Schiano et al., 2010) led to the hypothesis that Hfq and small RNAs may be involved in the regulation of T3SS in *Y. pestis* (Schiano et al., 2014). RNA sequencing led to the identification of 63 previously unidentified sRNAs. One of those sRNA, the *Yersinia*-specific sRNA, Ysr141, was encoded on the T3SS plasmid pCD1. In this study the authors demonstrated post-transcriptional activation of *Y. pestis yopJ*, via direct interaction of Ysr141 with the *yopJ* 5′UTR (Schiano et al., 2014).

### Quorum sensing

Quorum sensing is the regulation of gene expression in a population or cell density-dependent manner. Small RNA regulation of quorum sensing has been well-characterized in *Vibrio harveyi* and *Vibrio cholerae*. Global regulators of quorum sensing in *V. fischeri* LuxR and LuxI are homologous to YenR and YenI proteins of *Y. enterocolitica* (Tsai and Winans, 2011). These authors could demonstrate a role for YenR in quorum sensing and swarming motility. YenS, a 169 nt YenR-regulated sRNA, post-transcriptionally regulates expression of the *yenI* gene that encodes for a protein responsible for the synthesis of the 3-oxohexanoylhomoserine lactone (OHHL) pheromone (Tsai and Winans, 2011). Epistatic analyses of *yenI, yenR*, and *yenS* mutations, on swarming motility demonstrated that *yenS* mutants suppressed the hypermotility seen in *yenI* mutants (Tsai and Winans, 2011). This suggests that, in addition to post-transcriptionally inhibiting *yenI* expression, *yenS* directly stimulates swarming motility of *Y. enterocolitica* downstream of *yenI* (Tsai and Winans, 2011).

### Csr sRNAs

Csr sRNAs, along with 6S RNA, are amongst the most distinct non-Hfq dependent small RNAs in *E. coli*. These particular non-Hfq dependent small RNAs exert their regulatory function via direct binding and sequestration of the target proteins. There are two major Csr RNAs in *E. coli*, CsrB, and CsrC (Liu et al., 1997; Weilbacher et al., 2003). In *E. coli* CsrB and CsrC exert their regulatory effect by binding to, and thereby preventing the regulatory action of CsrA (Liu et al., 1997; Weilbacher et al., 2003). CsrA is a post-transcriptional regulator of genes necessary for carbon metabolism, motility, and biofilm formation in *E. coli* and other bacteria.

*Y. pseudotuberculosis* has a functional Csr regulatory system, including CsrB and CsrC sRNAs, involved in virulence (Heroven et al., 2008, 2012a; Table 3). CsrB and CsrC levels are differentially regulated based on genetic and environmental conditions, with CsrC levels elevated in complex media and absent in minimal media (Heroven et al., 2008). CsrB levels, which or normally low, are activated by the response regulator UvrY (Heroven et al., 2008). As already mentioned, Crp regulates the expression of the Csr RNAs (Heroven et al., 2012b). The Crp regulation of RovM-RovA-InvA regulatory cascade is through the Csr RNAs (Heroven et al., 2012b; Figure 3). Crp regulation of flagellar synthesis genes, *flhDC*, is through CsrA (Heroven et al., 2012b). CsrB and CsrC levels are repressed in *Y. pestis* isolated from the lungs of a mouse model of infection, suggesting that CsrB and CsrC expression is detrimental for virulence (Yan et al., 2013). The PhoP response regulator that senses environmental Mg^2+^, low pH, and antimicrobial peptides, through direct interaction between PhoP and the CsrC upstream region, also stimulates *csrC* transcription (Nuss et al., 2014; Figure 3). The stability of CsrC is variable between strains of *Y. pseudotuberculosis* YPIII and IP32953 due to the presence of a 20 nucleotide insertion mutation in the IP32953 *csrC* gene (Nuss et al., 2014). The *Y. pseudotuberculosis* IP32953 CsrC is significantly less stable than the YPIII CsrC, with t_1∕2_ of 90.5 and 41.7 min, respectively. When *Y. pseudotuberculosis* YPIII *csrC* gene was engineered to include the 20 nucleotide insertion from the *Y. pseudotuberculosis* IP32953 *csrC* gene, the YPIII CsrC transcript was destabilized to levels seen in IP32953 (Nuss et al., 2014).

As described in earlier sections, RovA is a premier global regulator of virulence gene expression in *Y. pseudotuberculosis* (Nagel et al., 2001; Figure 3). *Y. pseudotuberculosis* CsrA overexpression leads to the repression of RovA. CsrA plays a role in motility in both *Y. pseudotuberculosis* and *Y. enterocolitica* (Heroven et al., 2008; Legrand et al., 2015). RNA sequencing analyses of *Y. pseudotuberculosis* obtained using a mouse model of persistent infection revealed an overlap between genes modulated during persistent infection and the Crp/CsrA/RovA regulons, suggesting a role for CsrA in *Y. pseudotuberculosis* persistent infections (Avican et al., 2015). CsrA plays a role in resistance to several osmolytes (NaCl, KCl, CaCl_2_, and Rhamnose), temperature extremes (4 and 42°C), and antibiotic susceptibility (ampicillin and spectinomycin; Legrand et al., 2015).

## Potential targets for anti-bacterial development

Infectious disease is a major cause of mortality world-wide, and is now exacerbated by the rapid spread of antibiotic resistance. New approaches to treat infectious diseases are urgently needed, but persisting with traditional bacteriocidal antibiotics is pointless since bacteria are too adept at rapidly evolving resistance mechanisms toward them. One new approach being investigated to potentially limit mechanisms selecting for drug resistant bacteria is to identify bacteriostatic molecules that neutralize classical pathogen virulence factors or the mechanisms controlling their synthesis—the so-called “anti-infective” molecules (National Research Council, 2006). Many examples exist of high throughput screens designed to identify inhibitory molecules derived from natural or chemical libraries. In fact, studies using *Yersinia* as a model pathogen have demonstrated a potentially useful broad spectrum therapeutic target to be regulatory, assembly or functional mechanisms associated with the Ysc-Yop T3SS or the activity of its cargo (Kauppi et al., 2003; Tautz et al., 2005; Kim et al., 2009; Pan et al., 2009; Harmon et al., 2010; Wang et al., 2011; Jessen et al., 2014). This proved that identifying chemical inhibitors of classical virulence determinants (*i.e*.: anti-infectives) is a feasible approach to novel antibacterial innovation.

Moreover, ongoing methodological developments should benefit our future knowledge of the complex regulation of metabolism and virulence that underscores bacterial fitness during host infections. Greater understanding of these regulatory mechanisms will heighten selection of the most appropriate targets for the design and development of alternative novel anti-bacterials. For example, reduced *in vivo* fitness of several *Yersinia* mutants defective in metabolic pathways and transition metal transport pathways suggests that similar effects could be generated with the use of inhibitory anti-infectives preventing a pathogens quest for available carbon and trace element supplies (Klein and Lewinson, 2011; Palace et al., 2014; Pradel et al., 2014; Avican et al., 2015; Deng et al., 2015). A recent study has also successfully demonstrated the potential for targeting the CpxA-CpxR two-component regulatory system as a means to de-weaponize bacterial pathogens (Van Rensburg et al., 2015), which is consistent with our own observations that CpxA mutants deficient in phosphatase activity, exhibit over-active CpxA-CpxR signaling, and this leads to a dramatic reduction in virulence gene expression by *Y. pseudotuberculosis* (Carlsson et al., 2007a,b; Liu et al., 2011, 2012). Signaling molecules in the form of c-di-GMP and (p)ppGpp have also been the target for novel therapies against infectious disease. As these molecules have profound effects on bacterial pathophysiology, the synthesis of membrane permeable analogs might serve as useful antibacterial agents (Kalia et al., 2013). Much investment is still needed to uncover effective novel anti-infectives for use in the clinic, but studies on model bacterial systems such as pathogenic *Yersinia* give clear indication that new methods of infectious disease control will be discovered once again.

## Author contributions

SC, KT, and MF designed, drafted and revised the manuscript, and approved of its final content. SC, KT, and MF agree to be accountable for all aspects of the content.

### Conflict of interest statement

The authors declare that the research was conducted in the absence of any commercial or financial relationships that could be construed as a potential conflict of interest.
